# OmNI: a modular open-source framework for interactive multi-omics data integration and visualization

**DOI:** 10.1093/nargab/lqaf206

**Published:** 2026-01-10

**Authors:** Grace Potter, Jacob A Beierle, Camron Bryant, Sadhna Phanse, Carl White, Andrew Emili, Indranil Paul

**Affiliations:** Program in Quantitative Oncology, Division of Oncological Sciences, Knight Cancer Institute, Oregon Health and Science University, OR 97201, United States; The Jackson Laboratory, Research, 600 Main Street, Bar Harbor, ME 04609,United States; Laboratory of Addiction Genetics, Center for Drug Discovery, Department of Pharmaceutical Sciences, Northeastern University, MA 02115, United States; Program in Quantitative Oncology, Division of Oncological Sciences, Knight Cancer Institute, Oregon Health and Science University, OR 97201, United States; Program in Quantitative Oncology, Division of Oncological Sciences, Knight Cancer Institute, Oregon Health and Science University, OR 97201, United States; Program in Quantitative Oncology, Division of Oncological Sciences, Knight Cancer Institute, Oregon Health and Science University, OR 97201, United States; Program in Quantitative Oncology, Division of Oncological Sciences, Knight Cancer Institute, Oregon Health and Science University, OR 97201, United States

## Abstract

Omics Notebook Interactive (OmNI) is an R-based, open-source, and modular framework engineered for streamlined multi-omics data integration and analysis across diverse data types, incorporating interactive visualizations at each processing step. OmNI performs differential expression analysis utilizing customizable linear models, accommodating various covariates and complex experimental designs. For cross-omic layer integration, OmNI employs a modified *S*-score statistic, ensuring sensitive detection of differential features. The framework also integrates network and metabolomics data, offering detailed insights into regulatory mechanisms through comprehensive enrichment analysis using multiple pathway databases. Outputs include interactive HTML reports, CSV/TSV files, and Cytoscape-compatible objects. OmNI is readily deployable in both local and high-performance computing environments, enabling scalable data processing. Acknowledging the public health concerns of opioids, we performed TMT18-based deep proteome and phosphoproteome analysis of brains from genetically diverse collaborative cross diversity outbred (CC/DO) founder mouse strains exposed to fentanyl to demonstrate OmNI’s capabilities. The integrative *S*-score uniquely identified differential signaling and interaction hubs conserved across all strains and revealed strain-specific molecular neuro-responses to fentanyl. OmNI is freely available for download at https://github.com/gracerhpotter/OmNI and is also accessible via a web interface at https://emili-laboratory.shinyapps.io/omni/.

## Introduction

In recent years, advancements in omics profiling technologies have transformed biological research, enabling unprecedented insights into the molecular networks underlying cellular function. Mass spectrometry (MS)-based proteomics and metabolomics, along with transcriptomics, now provide comprehensive quantitative assessments of biomolecules across a wide range of experimental designs—from cell culture experiments to tissue biopsies and large collections of clinical samples [1]. While improved instrumentation and data acquisition techniques have greatly expanded the scope and utility of these methods, translating raw multi-omics data into actionable biological insights remains a formidable challenge. The sheer volume, heterogeneity, and complexity of multi-omics datasets demand sophisticated computational strategies for integration, interpretation, and visualization. Current bioinformatics tools often operate *in silos*, requiring expertise in multiple platforms, each with distinct operational requirements. This fragmentation not only increases the analytical burden but also limits accessibility, particularly for researchers lacking advanced computational and statistics training. As a result, many laboratories struggle to extract meaningful insights from their data, ultimately impeding the broader adoption and impact of multi-omics approaches in biomedical research [2].

A major bottleneck lies in the fragmented landscape of bioinformatics tools. Often, platforms tailored to single-omic analyses—proteomics, transcriptomics, or metabolomics—operate in isolation. This siloed nature complicates the integration of heterogeneous datasets and discourages routine multi-omics approaches. Further, many existing solutions require extensive programming expertise, limiting accessibility. Researchers proficient in coding languages like R or Python may navigate these hurdles, but the time, effort, and computational resources required can be prohibitive. Even when technical expertise is available, standardizing workflows, ensuring reproducibility, and integrating data from different omics layers into a coherent and statistically valid analysis often involves juggling multiple tools and data formats, knowledge across multiple domains, and statistics [2]. Existing user-friendly prominent platforms like Perseus [3], STATegRa [4], OmicsNet [5], PaintOmics [6], and NetworkAnalyst [7] address some challenges but can be limited by input format constraints, specific platform requirements, lack of advanced analytical features, or insufficient support for end-to-end integrative analyses.

To overcome these barriers in multi-omics research, we have developed Omics Notebook Interactive (OmNI)—an R-based, open-source, user-friendly platform designed to streamline end-to-end multi-omic data analysis workflows, from data integration to actionable biological insights, within a single, reproducible environment. OmNI accepts inputs from major proteomics platforms (e.g. MaxQuant, Proteome Discoverer, and FragPipe), as well as other generic expression matrices, ensuring broad compatibility with diverse data sources, including proteomic, transcriptomic, and metabolomic datasets. Once loaded, datasets undergo robust user-guided (with sensible default settings) preprocessing, pairing multiple normalization and imputation strategies with interactive visualizations, enabling transparent, data-driven decisions tailored to the dataset at hand. OmNI delivers statistical rigor via customizable linear models (e.g. covariates, contrasts, and time-series designs) powered by limma, facilitating robust differential expression, pathway enrichment analysis, and batch correction. A key strength of OmNI lies in its multi-omics integration capabilities, leveraging the *S*-score statistics [8] to integrate subtle but biologically meaningful signals across multiple omic layers—overcoming a key limitation of conventional single-omic approaches. OmNI leverages the strength of network analysis by implementing the prize-collecting Steiner forest (PCSF) algorithm [9] to map differential features onto a high-quality protein interaction network to uncover context-specific hidden regulatory mechanisms. OmNI offers a comprehensive enrichment analysis suite, extensive visualization options, and an automated reporting engine that generates interactive HTML outputs—complete with interactive plots and tables. OmNI operates efficiently in both local and high-performance computing environments, democratizing access to advanced multi-omics analysis for novices and experts alike.

The opioid crisis persists as a major public health challenge, with fentanyl’s extreme potency and widespread availability driving overdose deaths [10]. To elucidate the genetic underpinnings of molecular responses to fentanyl, we utilized the genetically diverse mouse collaborative cross diversity outbred (CC/DO) founder panel [11], applying high-resolution deep proteomics and phosphoproteomics to brain tissue. This study serves as a rigorous case example, showcasing OmNI’s capabilities in multi-omic data analysis, even with challenging, noisy, and genetically and phenotypically heterogeneous datasets. We first detail OmNI’s flexible preprocessing pipeline, covering data import, quality control, missing value handling, and normalization method evaluation. Subsequently, we demonstrate its robust differential expression analysis using limma, followed by multi-omics integration via S-scores. Our analysis reveals both conserved physiological responses to acute fentanyl exposure and distinct strain-specific signaling, including differential protein expression interaction networks linked to opioid sensitivity. This case study guides the reader through OmNI’s workflow, demonstrating its capacity to extract profound biological insights from complex multi-omics data.

In summary, OmNI represents a powerful and versatile tool for integrative multi-omics analysis. Its flexible, open-source, R-based framework facilitates broad adoption, enabling researchers across expertise levels to fully leverage their multi-omics datasets. By bridging the gap between diverse omics technologies and unifying analytical workflows into a streamlined, all-in-one platform, OmNI could facilitate data integration, lowering computational barriers and accelerating the discovery of meaningful biological insights.

## Materials and methods

### Open-source framework

OmNI’s flexible, open-source framework is designed to empower researchers of varying expertise levels to harness the full potential of their multi-omics datasets. The software architecture is modular and containerized, ensuring scalability and reproducibility. This framework comprises three main components:

#### Core structure

Built on the R Shiny framework, OmNI integrates diverse analytical functions into a centralized interactive interface. The core scripts coordinate module interactions with user and data inputs, managing global parameters, configurations, and data input/output specifications. This design facilitates customizable application of modular scripts and controls workflow execution. The modules perform the bulk of OmNI’s statistical processing and data visualization tasks. Starting with preprocessing, normalization, and quality control, the modules handle linear modeling, pathway enrichment, network analysis, integration, and report generation based on interactive user inputs. This modular design promotes flexibility and ease of maintenance, allowing for ongoing development and adaptation to emerging analytical needs.

#### Implementation stack

The analytical engine is implemented in R (v4.1+), utilizing the Shiny package for the interactive application. Data handling and manipulation are facilitated by packages such as tidyverse and Biobase. Statistical analyses, including modeling and imputation, are conducted using limma, stats, edgeR, pcaMethods, and NormalyzerDE. Visualization capabilities are powered by ggplot2, plotly, Glimma, and ComplexHeatmap. Enrichment analyses are performed using clusterProfiler, while network analyses and visualizations leverage igraph, influential, and visNetwork, with PCSF algorithms facilitating context-specific interaction network mapping (Table 1).

#### Interactivity

OmNI’s user interface supports and encourages, highly interactive analytical workflows. However, users can choose their level of engagement with the analysis, from a detailed, step-by-step exploration of each visualization to a streamlined, hands-off experience ideal for non-bioinformaticians or those seeking a quick overview of their data. Sensible defaults have been used throughout the analyses in line with standard practices.

#### Report generation and sharing

OmNI generates comprehensive, publication-ready reports using R Markdown, delivering a rich data analysis summary in an interactive HTML format. This dynamic report seamlessly integrates interactive data visualizations within a single, shareable file, facilitating easy exploration and interpretation of results. Beyond the interactive HTML report, OmNI also generates a structured output folder containing publication-quality PDF Figs, readily accessible data tables, and files formatted for seamless integration with external bioinformatics tools. For example, enrichment summaries are specifically formatted for direct input into Cytoscape, enabling immediate network visualization and analysis. This multi-faceted reporting approach ensures both accessibility and reproducibility of results, streamlining the process from initial analysis to publication-ready Figs and Supplementary data.

### Data input and processing

OmNI’s data processing implementation offers a robust and flexible framework for multi-omics data analysis, integrating comprehensive input processing, quality control, and statistical analysis to facilitate actionable biological insights.

#### Data inputs

OmNI supports multiple input formats through dedicated parsers, accommodating output files from MaxQuant, FragPipe, Proteome Discoverer, MetaboAnalyst, and generic sample-feature matrices. An accompanying annotation file provides context for sample grouping and specifies intensity columns for simultaneous processing of multiple files. The data input interface offers real-time format validation and informative error messages for misformatted inputs. Subsequent data processing includes filtering, preprocessing, and normalization based on user-defined parameters. Normalization methods address batch effects, and users can selectively exclude outlier samples to enhance data quality.

#### Quality control

Initial preprocessing involves the removal of annotated contaminants and reverse sequences, as well as filtering out rows that do not meet a user-defined threshold for missing values. Cleaned data are log-transformed, annotated with UniProt identifiers, and subjected to normalization. Available normalization techniques include quantile, loess, median, medianMAD, variance stabilizing normalization (VSN), z-transformation, and internal reference scaling, allowing users to select the method best suited to their dataset. Optional missing value imputation is performed using nearest neighbor approaches via local least squares from the pcaMethods package (Table 1).

#### Differential analysis

OmNI leverages the powerful limma package to robustly identify differentially expressed genes, even in complex experimental settings. By employing linear models and empirical Bayes shrinkage, limma effectively handles confounding factors, intricate study designs, and limited sample sizes. OmNI provides a user-friendly interface to precisely specify covariates, interactions, batch effects, and blocking structures directly within the annotation file.

#### Pathway enrichment analysis

OmNI provides a comprehensive and highly configurable framework for pathway enrichment analysis, allowing researchers to explore biological processes in detail. The platform leverages the clusterProfiler package, offering both gene set enrichment analysis (GSEA) and over-representation analysis (ORA) to cater to different analytical approaches. GSEA allows for the assessment of pathway enrichment by considering the ranking of all genes in the dataset, while ORA identifies significantly over-represented pathways based on a predefined gene list. Users can select from a diverse range of pathway databases, including MSigDB, KEGG, Reactome, and Gene Ontology (GO), enabling analysis tailored to specific biological domains.

#### Visualization

Exploration and manipulation of regression model outputs are facilitated through various tools, including volcano plots, Glimma interactive *XY* plots, ranked or unranked enrichment analyses, and network analyses. For example, principal component analysis (PCA) comparisons before and after covariate inclusion in the linear model could help in evaluating the impact of these variables on the regression model. These features enhance the interpretability of the data and support comprehensive downstream analysis.

### Multi-omics integration using *S*-scores

For integrative analysis, we adopted the methodology described by Balbin *et al.* [8]. The *S*-score is a statistical measure that integrates multiple omics data types, providing a unified significance score at the gene level.

#### Normalization and weighting

The first step involves normalization and weighting of features within each dataset. For each feature (gene, protein, phosphosite, etc.) denoted by *i* in dataset *j*, the log fold change (logFC) value was transformed into a *z*-score (*z*_i_) using the following formula:


\begin{eqnarray*}
{z_i} = \frac{{\textrm{logF}{C_{ij}} - {\mu_j}}}{{{\sigma _j}}},
\end{eqnarray*}

where

- *μ_j_* is the mean logFC value in dataset *j*,
- σ_*j*_ is the standard deviation of logFC values in dataset *j*.

*Z*-scores center and standardize the data within each dataset, allowing for comparisons across datasets with different measurement units. Next, a weight (*w*_*i*_) is assigned to each feature based on the inverse square root of the number of observations (*N*_*j*_) in its dataset:


\begin{eqnarray*}
{w_i} = \frac{1}{{\sqrt {{N_j}} }}.
\end{eqnarray*}

This weighting scheme ensures a fairer comparison across features within datasets of varying sizes. The weighted *z*-score (*wz*_*i*_) for each feature is then calculated by multiplying the *z*-score (*z*_*i*_) by its corresponding weight (*w*_*i*_):


\begin{eqnarray*}
w{z_i}\; = \;{z_i}\;\times\;{w_i}.
\end{eqnarray*}

#### Combining *z*-scores

After a full join on gene identifiers which creates a comprehensive dataset that includes all feature alterations; for each gene, a combined weighted *z*-score (comb_*wz*_*i*_) is calculated by summing the weighted *z*-scores (*wz*_*i*_) across all relevant datasets (*j*_1…*n*_):


\begin{eqnarray*}
{\mathrm{comb}}\_w{z_i}\; = \mathop \sum \limits_{{j_1}}^{{j_n}} w{z_i}.
\end{eqnarray*}

A combined weight (comb_*w*_*k*_) is then computed as the square root of the sum of squared individual weights (*w*_*i*_) from all datasets:


\begin{eqnarray*}
{\mathrm{ comb}}\_{w_k}\; = \;\sqrt {\mathop \sum \limits_{{j_1}}^{{j_n}} } w_i^2.
\end{eqnarray*}

#### *S*-score calculation

The *S*-score is derived by normalizing the combined weighted *z*-score (comb_*wz*_*i*_) by the combined weight (comb_*w*_*k*_):


\begin{eqnarray*}
S \hbox{-} \textit{score}\; = \frac{{{\mathrm{ comb}}\_w{z_i}\;}}{{{\mathrm{ comb}}\_w_{k}\;}}.
\end{eqnarray*}

This normalization step ensures the *S*-score remains on a standard scale even when the number of datasets being integrated changes.

#### Significance testing

*P*-values (sscore_pval) are computed using the standard normal cumulative distribution function, adjusted for two-tailed tests. This assesses the statistical significance of the *S*-score. Finally, the Benjamini–Hochberg procedure is used to adjust *P*-values for multiple testing (sscore_adj_pval), controlling the false discovery rate (FDR).

### Network analysis

#### Prize-collecting Steiner forest

We utilized the PCSF algorithm for network analysis, as outlined by Akhmedov *et al.* [9]. This algorithm is particularly suited for identifying relevant subnetworks within larger interaction networks, considering both the connectivity between important nodes (terminals) and the cost of including additional (Steiner) nodes. In our implementation, we leverage the computed *S*-scores as node prizes [p(v)] within the PCSF framework. The network is represented as a graph G = (V, E), where V is the set of nodes (genes) and E is the set of edges (interactions between nodes). Each edge e ∈ E incurs a cost [c(e)] based on the corresponding interaction’s experimental confidence derived from the STRING database [12]. Interactions with a combined score of ≥ 0.7 were used to ensure high-confidence interactions. The PCSF algorithm aims to find a subgraph G' = (V', E') that maximizes the sum of the prizes collected from the included nodes [∑(v ∈ V') p(v)] while minimizing the total edge cost [λ ∑(e ∈ E') c(e)]. Here, λ is a tuning parameter that balances the trade-off between node importance (reflected by *S*-scores) and edge costs (representing interaction confidence). This optimization problem can be formulated as


\begin{eqnarray*}
\mathrm{ {{ maximize}}\;\Sigma\!\left( {v\in V^{\prime}} \right)\,p\!\left( v \right) - \lambda \Sigma\!\left( {e\in E^{\prime}} \right)\,c\!\left( e \right)}.
\end{eqnarray*}

The resulting subnetwork, enriched with high-scoring nodes (terminals) and high-confidence interactions, is likely to capture the most significant biological pathways and interactions, providing valuable insights into the underlying biological mechanisms.

### Diversity outbred founder strain experiment

Female mice from the panel of inbred strains comprising the collaborative cross diversity outbred (CC/DO) strains as well as DBA/2J (8 weeks old at time of arrival) from nine genetically diverse strains (A/J, DBA/2J, 129S1/SvImJ, NOD/ShiLtJ, NZO/HlLtJ, C57BL/6J, PWK/PhJ, CAST/EiJ, and WSB/EiJ) [11] were housed two per cage under controlled temperature (22°C ± 1°C) and a 12:12-h light/dark cycle. All procedures were approved by the Institutional Animal Care and Use Committee (IACUC protocol #15326) at Boston University and carried out in the laboratory of Prof. Camron Bryant in accordance with institutional guidelines. The study was conducted over three consecutive days. On Days 1 and 2 (D1, D2), mice were acclimated to the testing apparatus following intraperitoneal (i.p.) administration of saline and observed for 30 min. On Day 3 (D3), mice received either fentanyl (0.2 mg/kg, i.p.) or volume-matched saline (*n *= 2 per strain/treatment group). Mice were then sacrificed at 10 min post-injection by cervical dislocation. Whole brains were rapidly dissected, rinsed in ice-cold phosphate buffered saline, flash-frozen in a dry ice/isopentane slurry, and stored at –80°C until further analysis. Fentanyl (Sigma–Aldrich) was prepared in sterile saline and administered intraperitoneally at a dose of 0.2 mg/kg in a volume of 10 ml/kg.

### Mass spectrometry

#### Protein extraction, TMT labeling, fractionation, and phosphoproteomic enrichment

Protein extracts from flash-frozen brain tissues were prepared using the GuHCl method as described previously [13]. One hundred micrograms of protein per sample were digested overnight at 37°C with sequencing-grade trypsin (Promega) at a 1:50 enzyme-to-protein ratio. Digested peptides were labeled with TMTpro 18-plex reagents (Thermo Fisher Scientific) according to the manufacturer’s protocol. Briefly, peptides were resuspended in 100 mM HEPES (pH 8.5), mixed with TMT reagents dissolved in anhydrous acetonitrile (ACN), and incubated at room temperature for 1 h. The reaction was quenched with 5% hydroxylamine, and labeled peptides were pooled, desalted using C18 solid-phase extraction cartridges (Waters), and dried under vacuum. For proteomic analysis, the pooled TMT-labeled peptides were fractionated using high-pH reverse-phase chromatography on an XBridge BEH C18 column (Waters) with a 10%–40% ACN gradient over 60 min. A total of 96 fractions were collected and concatenated into 24 fractions. After aliquoting out 5% for proteome analysis, the remaining were concatenated into 12 fractions for phosphopeptide enrichment using Fe-NTA magnetic beads (Thermo Fisher Scientific) according to the manufacturer’s protocol. All peptides were dried and resuspended in 1% formic acid for LC-MS/MS analysis.

#### LC-MS/MS analysis

Fractionated peptides were analyzed on an Exploris 480 mass spectrometer (Thermo Fisher Scientific) coupled to a NEO Vanquish UHPLC system (Thermo Fisher Scientific). Peptides were separated on a PepMap RSLC C18 column (75 µm × 50 cm, 2 µm particle size) using a 120-min gradient of 2%–30% ACN in 0.1% formic acid at a flow rate of 300 nl/min. MS1 spectra were acquired at 120 000 resolution (*m*/*z* 200) with an AGC target of 3e6 and a maximum injection time of 50 ms. MS2 spectra were acquired in data-dependent acquisition (DDA) mode at 45 000 resolution (*m/z* 200) with an AGC target of 1e5, a maximum injection time of 86 ms, and a normalized collision energy of 32%. Dynamic exclusion was set to 30 s.

#### Data processing

Raw MS files were processed using FragPipe (v18.0) with the MSFragger search engine. Spectra were searched against the UniProt *Mus musculus* reference proteome (downloaded March 2023) with common contaminants appended. Search parameters included a precursor mass tolerance of 20 ppm, fragment mass tolerance of 0.05 Da, fixed TMTpro modifications on lysine residues and peptide N-termini, variable modifications for methionine oxidation and phosphorylation (S/T/Y), and a maximum of two missed cleavages. Peptide-spectrum matches were filtered at a 1% FDR using the Philosopher tool within FragPipe. TMT reporter ion intensities were normalized and quantified using the IonQuant module.

## Results

### OmNI’s modular architecture and guided interactive workflow

**Figure 1.**
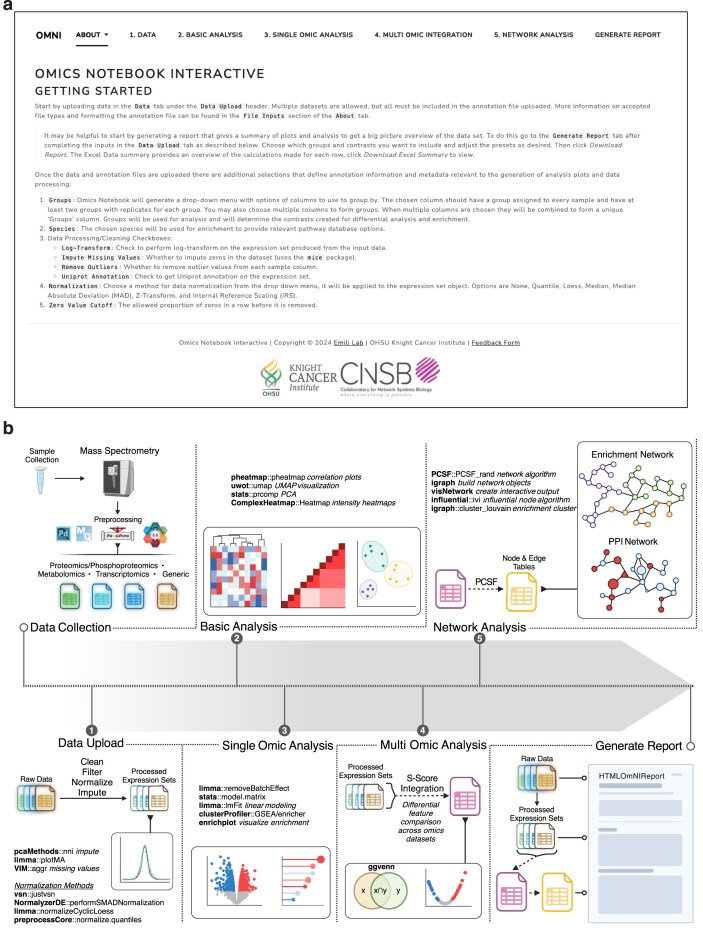
Overview of OmNI processing platform. (**a**) Screenshot of the OmNI’s landing page, showing the initial navigation interface numbered tabs with processing steps. (**b**) Schematic representation of OmNI’s interactive pipeline for multi-omics data analysis, including inputs, processing modules and packages, and visualization options. Key features include enrichment analysis, multi-omic integration, and network analysis.

OmNI is a modular R-based platform designed to unify multi-omics data exploration, analysis, and interpretation within an intuitive, scalable interface (Fig. 1). Built on principles of modularity, interoperability, and reproducibility, OmNI integrates disparate omics data types (proteomics, phosphoproteomics, and metabolomics) into a cohesive analytical workflow. Its architecture separates core functionalities—data import, statistical modeling, integration, and visualization—into self-contained modules, enabling users to execute standalone analyses (e.g. quality control) or chain workflows (e.g. differential expression → pathway enrichment → network analysis). The back end utilizes established and regularly maintained CRAN/Bioconductor packages, ensuring efficient performance, while R-markdown reporting facilitates seamless data visualization and dissemination.

The OmNI interface (Fig. 1a) guides users through six sequential stages: “Data,” “Basic Analysis,” “Single-Omic Analysis,” “Multi-Omic Integration,” “Network Analysis,” and “Generate Report.” The sidebar offers context-sensitive parameter controls that dynamically adjust to each analytical stage, e.g. “Normalization” methods in “Data” (Supplementary Fig. S1a) and “Pathway databases” in “Enrichment” (Supplementary Fig. S1b). The central visualization pane renders interactive plots (e.g. PCA, heatmap, and volcano) and tables using Plotly and Glimma, allowing “hover-to-annotate” and zoom functionalities.

**Figure 2.**
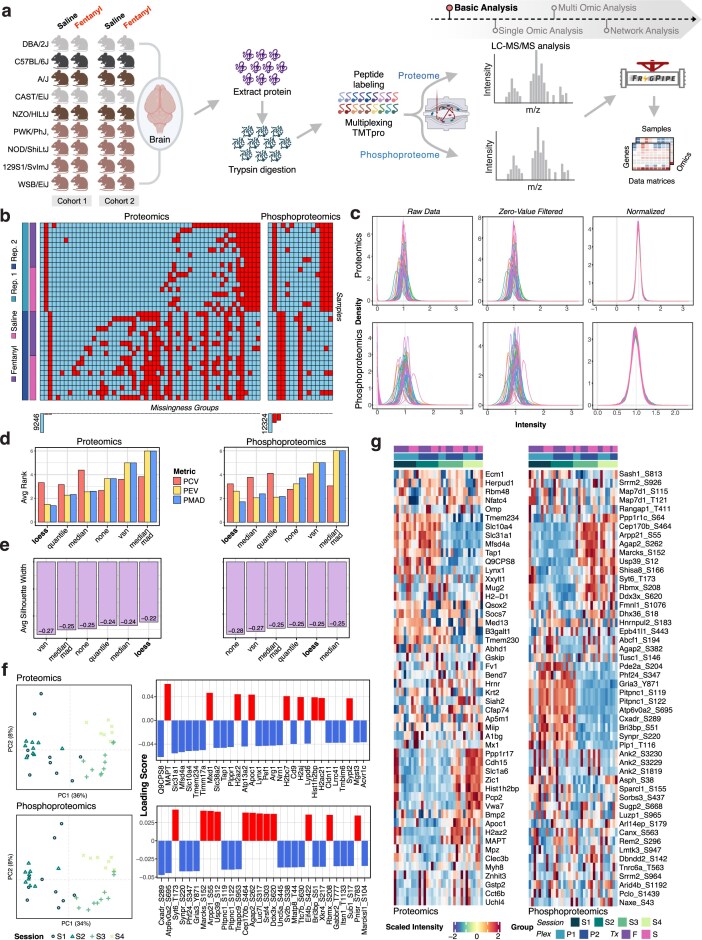
Evaluation and data quality assessment of proteomics and phosphoproteomics datasets. (**a**) Schematic representation of the experimental design, from sample conditions and processing to downstream analysis flow. (**b**) Missing value distribution across samples for both proteomics (left) and phosphoproteomics (right) datasets, indicating missingness across replicates prior to filtering. (**c**) Density plots showing intensity distributions before and after filtering and normalization for proteomics and phosphoproteomics datasets, demonstrating improved comparability across samples. (**d**) Normalization performance comparison using three evaluation metrics: pooled median absolute deviation (PMAD), pooled coefficient of variation (PCV), and pooled estimate of variance (PEV). (**e**) Normalization performance comparison using average silhouette width, where higher values indicate better clustering among distinct groups. (**f**) PCA of normalized data with a loading vectors bar plot showing separation of biological groups and contributing variables for both proteomics and phosphoproteomics. (**g**) Heatmaps of *z*-scored normalized intensities, with hierarchical clustering of samples by condition (top annotation), illustrating condition-specific expression patterns.

**Figure 3.**
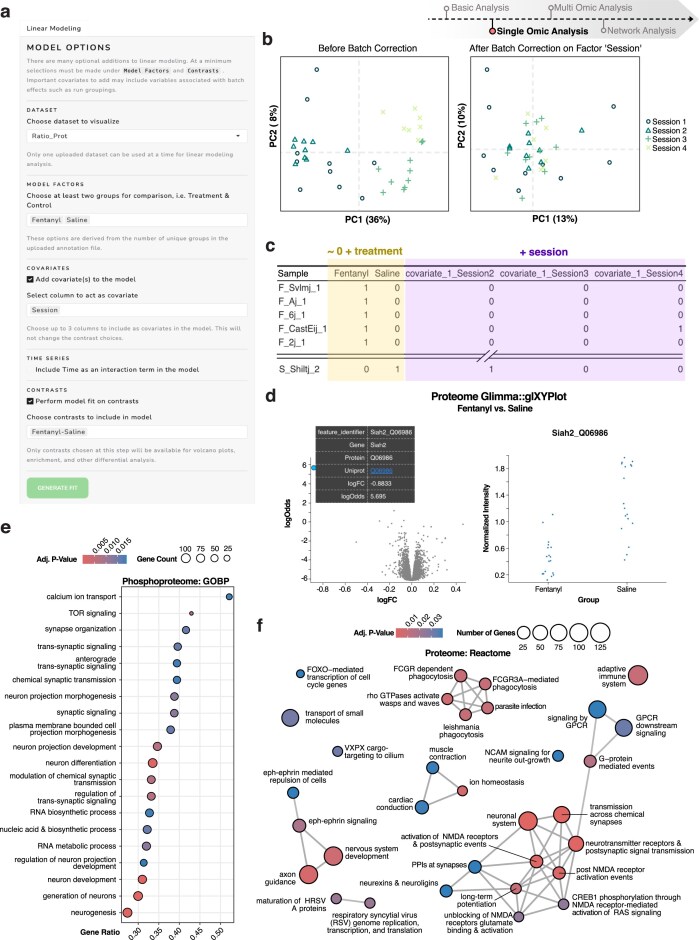
Visualization of linear model setup and functional interpretation of proteomic and phosphoproteomic analyses. (**a**) Screenshot of the user interface for specifying linear modeling parameters, including experimental factors, covariates, time series, and contrast definitions. (**b**) PCA plots showing intensity distributions before and after batch correction by the factor “Session,” demonstrating correction of systematic technical variation via limma modeling. (**c**) Table of the resulting model design matrix illustrating how experimental conditions and covariates are encoded for downstream analysis. (**d**) Differential expression outputs generated by Glimma::glXYPlot, including a volcano plot of log₂ fold change versus log-odds and a plot of normalized intensities by sample highlighting Siah2 as a significant feature. (**e**) Dot plot visualization of pathway enrichment for the phosphoproteome dataset using Gene Ontology Biological Process (GOBP) terms, with pathways ranked by statistical significance and effect size. (**f**) Enrichment map network visualization of differentially expressed proteins using Reactome pathway annotations, showing clustered biological themes.

OmNI’s analytical workflow (Fig. 1b) starts by importing raw feature-level data, accepting outputs from major proteomics platforms like MaxQuant [14] and FragPipe [15], or various metabolomics pipelines [16]. A generic feature-by-sample matrix can also be used as input. The initial data import and harmonization process includes preprocessing, guided by HGNC symbols and UniProt IDs, as well as contaminant filtering and log transformation. OmNI then provides users with the ability to test various normalization methods, leveraging comparative statistics and intuitive plots to identify the optimal approach for their dataset (e.g. medianMAD and VSN), and offers functionality for missing value imputation. This stage is fully equipped with interactive quality control (QC) visualizations—such as violin plots, PCA, and MD plots—which empower users to conduct exploratory analyses (e.g. PCA, UMAP, and correlation heatmaps) and make informed, data-driven decisions on critical parameters, such as the most suitable normalization method for the dataset (see Fig. 2). The QC-verified, normalized dataset and the annotation table are then passed to the limma framework for differential expression analysis, enabling robust identification of condition-specific features. The linear model can be constructed to account for confounders—covariates, batch effects, and other design factors—ensuring statistically sound inference. Model assumptions are thoroughly evaluated through diagnostic visualizations (see Fig. 3), affirming the integrity and reliability of the results.

**Figure 4.**
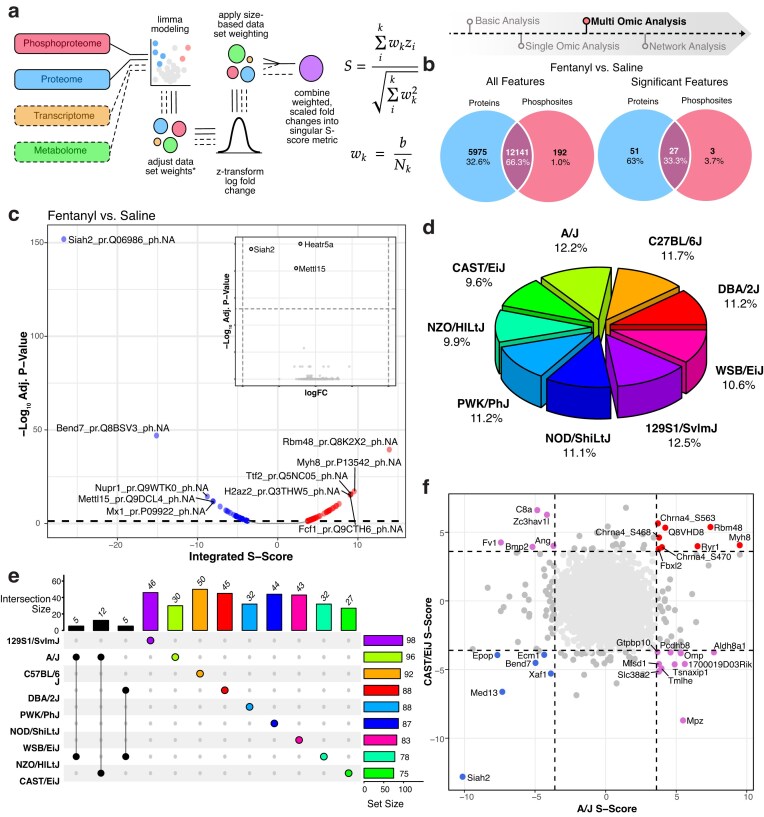
Multi-omic integration using the *S*-score framework reveals strain-specific and shared molecular responses. (**a**) Representative overview of the *S*-score statistical method for integrating multi-omic data, enabling combined assessment of differential expression across omic layers. (**b**) Venn diagrams showing the overlap and uniqueness of detected features in proteomic versus phosphoproteomic datasets, presented for both the entire dataset and the subset of differentially expressed (DE) features. (**c**) Volcano plot displaying integrated *S*-score versus adjusted *P*-value. An inset volcano plot in the upper right corner compares the fentanyl versus saline contrast before *S*-score integration, illustrating how integration enables detection of features previously missed. (**d**) Pie chart depicting the proportional contribution of each mouse strain to the set of significant *S*-score features, highlighting strain-specific biological signals. (**e**) UpSet plot showing the distribution of significant features across strains, including strain-specific features and shared overlaps, with the A/J and CAST/EiJ strains sharing 12 unique features, among the largest overlaps. (**f**) Dot plot comparing A/J and CAST/EiJ, the two strains with the greatest overlap in DE features. Features are colored by concordance or discordance in directionality, providing insight into shared and divergent biological responses.

**Figure 5.**
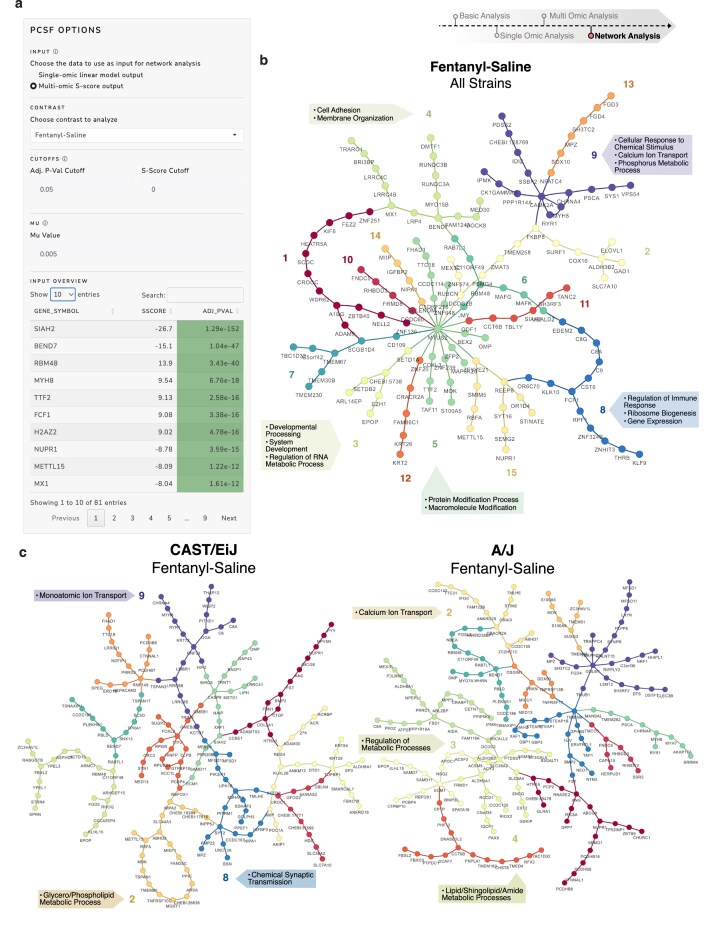
PCSF-based network construction and pathway cluster visualization for fentanyl response across strains. (**a**) Screenshot of the PCSF network visualization interface, illustrating user-defined options for input data type, node and edge cutoffs, and the *μ* parameter, which balances network connectivity and cost. (**b**) Cluster-enriched PCSF network for the broad fentanyl versus saline comparison across all mouse strains. Major clusters are annotated with representative GO Biological Process pathways, highlighting functionally enriched modules. (**c**) Strain-specific cluster-enriched PCSF network for the CAST/EiJ strain under the same comparison, revealing distinct and overlapping pathway clusters relative to the overall response. (**d**) Strain-specific cluster-enriched PCSF network for the A/J strain, visualized similarly, showing pathway enrichment patterns that differentiate it from both the full dataset and CAST/EiJ.

Integration of multiple layers of gene product expression is performed using a modified *S*-score method, converting limma-derived logFC values into dataset-weighted *z*-scores, which are then combined across datasets to generate unified, gene-level statistical measures. Significant features, with an adjusted *P*-value threshold of <0.05 (default, but user-configurable), are identified using the standard normal cumulative distribution function for two-tailed tests. The PCSF approach then reconstructs biologically relevant subnetworks using significant features (genes and metabolites) with *S*-scores as node prizes and a manually curated protein–protein and protein–metabolite interaction database as the scaffold (see Fig. 4). Results are compiled into interactive HTML reports, combining analytical provenance, visualizations, and exportable data (see Fig. 5). OmNI’s backend integrates CRAN and Bioconductor packages for omics-aware processing: limma for linear modeling, clusterProfiler for enrichment, and igraph for network analysis. Custom implementations include the *S*-score method for multi-omic integration and PCSF for context-specific subnetworks. All codes are written in R, open-source and could be freely accessed here at https://github.com/gracerhpotter/OmNI.

OmNI fills a crucial gap in multi-omics research by democratizing advanced analytical workflows without compromising rigor. Its modular design enables non-computational users to perform end-to-end analyses—from raw data to biological insights—while offering extensibility for developers. OmNI is built entirely in R, facilitating easy deployment. Its open-source and modular framework ensures seamless adaptability to emerging omics technologies, including single-cell and spatial omics.

### Data import, exploration, QC, and preprocessing

Despite its clinical importance in pain management, opioids underlie a major public health crisis, with fentanyl emerging as a primary driver of overdose deaths due to its extreme potency and widespread availability [17]. Genetic factors contribute to variation in physiological and behavioral response to opioids, including analgesia, respiratory depression (associated with overdose-related deaths), and susceptibility to opioid use disorder. Therefore, understanding genetic influences on the molecular mechanisms underlying fentanyl-induced neurobiological responses is critical. Diversity Outbred mice, which harbor a level of genetic diversity that mirrors human population variation, provide a powerful model to investigate genetic variation in the molecular responses to fentanyl exposure [11]. By employing high-resolution TMT18 multiplexed deep proteomics and phosphoproteomics profiling of brain tissue across nine CCF strains administered fentanyl for 10 min (the peak time point of behavioral action), we aimed to identify immediate molecular responses to this acute exposure (Fig. 2a). Here, we used this case study to guide the user through OmNI’s workflow and to showcase its capacity to democratize multi-omics analysis.

Step 1: Data import and harmonization: Raw MS data generated on the Thermo Exploris 480 platform were processed using FragPipe (FragPipe version 22.0, MSFragger version 4.1), with peptide-spectrum matching performed against the UniProt Mus musculus reference proteome (downloaded on October 2024). TMT reporter ion intensities were extracted from FragPipe’s “tmt-report” output, specifically using the “abundance_protein_none.tsv” file for protein-level and the “abundance_multi-site_none.tsv” file for phosphosite-level quantifications. An accompanying “experiment_annotation.tsv” file contained sample-level metadata, including TMT-channel, strain, treatment (fentanyl or saline), biological replicate, and session, with support for user-defined variables of interest. These raw datasets, comprising 9942 proteins and 22 573 phosphosites derived from brain tissue, were imported into OmNI for structured multi-omics integration and downstream analysis. OmNI’s built-in feature harmonization framework mapped species-aware UniProt accession IDs to standardized gene symbols and incorporated functional annotations such as GO terms and UniProt functions and disease associations. This harmonization enabled consistent feature labeling across omics layers and ensured interpretability of the results. Internally, OmNI restructures the imported data into standardized “ExpressionSet” objects, which contain quantification matrices alongside corresponding feature- and sample-level metadata. This standardized data architecture forms the backbone for downstream analyses across the multiple omics’ layers, supporting inter-modular operability, e.g., normalization, statistical modeling, and biological interpretation.
Step 2: Quality control and missingness assessment: OmNI equips users to assess data quality through its preprocessing module, starting with a global heatmap of missingness patterns across samples (Fig. 2b). Using our example dataset from the DO/CC founder panel, this heatmap reveals sample-wise dropouts, guiding users in evaluating their own data’s integrity. Owing to the presence of missing values, we applied a ≥30% (user-defined) missing value filter retaining 9287 proteomic and 12 333 phosphoproteomic features. Users can inspect pre-normalization intensity distributions via density (Fig. 2c) and violin plots (Supplementary Fig. S2a), which here showed no technical bias. Because the heatmaps and intensity distributions confirmed no outliers or dropouts, no samples were removed. The heatmap also highlighted TMT-plex-dependent missingness without sample-specific patterns, suggesting missing-at-random (MAR). This effect, common in TMT-labeled multiplexed DDA datasets [18], illustrates how OmNI assists users in identifying and addressing such systematic artifacts.
Step 3: Data normalization and missing value imputation: OmNI provides users with options for testing multiple normalization methods supported by diagnostic plots to tailor analysis to their data. Normalization corrects systematic biases, ensuring reliable statistical analysis like linear modeling. Users can expect consistent total intensities between the samples unless biological factors (e.g. knockouts and drug treatments), specific experimental design (e.g. pull-downs), or technical issues (e.g. inconsistent sample preparation, instrument drift, or poor ionization) skew results. In label-free designs, run-to-run stochastic variations, while in TMT workflows, batch effects can amplify differences, necessitating proper management of failed samples and robust normalization. High intragroup consistency and low intergroup overlap enhance the sensitivity for detecting genuine biological signals across conditions. Variation metrics (PCV, PEV, PMAD) (Fig. 2d) and silhouette plot (Fig. 2e) demonstrated that “loess” normalization best minimized intragroup variation while preserving intergroup differences. Guided by the above diagnostic plots, we also opted against imputation given the minimal missingness in our two-replicate TMT-plex design. While imputation is optional (e.g. limma can inherently accommodate missing data), users may choose to impute values—after applying appropriate missing value filters—if supported by their experimental context. Imputation may be warranted in cases where the remaining missingness is primarily technical (e.g. due to stochastic peptide identification across runs), when downstream analyses require complete data matrices (e.g. in certain machine learning applications), or when missing values reflect true biological absences (e.g. in immunoprecipitation experiments where IgG control samples are expected to be missing true interactors). However, the choice of imputation method should carefully consider the nature of the missingness and the experimental context.
Step 4: Exploratory data analysis. At this step of the analysis, OmNI”s interactive visualization suite provides complementary methods for in-depth data exploration. For example, the PCA plot in Fig. 2f clearly shows grouping by “session,” but not by “treatment,” “replicate,” or “TMT” batch (Supplementary Fig. S2b). This pattern suggests that the genetic diversity of the mice has a stronger influence on the PCA groupings than the acute 10-min fentanyl exposure, which is unlikely to globally alter the brain proteome. UMAP plots (perplexity = 15) in Supplementary Fig. S2c further reinforce these observations.

The PCA loadings (Fig. 2f, right panel) identify features most responsible for variation, while heatmaps of the top 10% (user-defined) most variable features (Fig. 2g) reveal expression patterns, clustering, and condition-specific responses across samples. Mean-difference (MD) plots (Supplementary Fig. S2d), which display log fold changes against average expression, further illustrate differential feature abundance between fentanyl versus saline comparison. Users can utilize these plots to begin to uncover biological insights in their own studies. In our CC dataset, samples are grouped by “session,” leading us to employ OmNI’s advanced linear modeling capabilities to identify differential features of interest.

To summarize, the exploratory analyses described in this section, enabled by OmNI’s visualization toolkit, provide critical preliminary insights prior to formal statistical modeling:

1. OmNI’s diagnostic plots (e.g. density/violin plots, sample dropouts, and outlier detection) flag potential technical anomalies. In our CC dataset, no extreme outliers were detected, ensuring robust cohort-wide conclusions.
2. Systematic biases were successfully corrected through appropriate normalization. Given the minimal missingness of MAR type, imputation was deemed unnecessary.
3. PCA/UMAP revealed session-driven clustering, guiding our inclusion of “session” as a covariate in linear models to isolate biological variation mediated by genetics and fentanyl treatment.
4. The MD plot further confirmed the successful capture of fentanyl induced changes, particularly evident among kinases and synaptic proteins, which are known to be key regulators of opioid response.

Overall, OmNI’s integrated toolkit streamlines preprocessing. Its diagnostic workflows ensure data quality (outlier detection, bias correction via data-driven normalization), while dimensionality reduction (PCA/UMAP) identifies confounding variables for modeling. Heatmaps and MD plots rapidly surface perturbations in functionally relevant proteins, and the pipeline robustly converts raw data into an analysis-ready matrix—resolving technical artifacts without masking biological signals.

### Streamlined statistical modeling with limma for complex experimental designs

OmNI streamlines complex statistical workflows through a user-friendly interface that integrates the limma framework, renowned for its robust capabilities in multifactorial linear modeling, empirical Bayes moderation, and pathway enrichment—all without requiring coding expertise (Fig. 3a). Designed for rigorous hypothesis testing in diverse and complex experimental designs, OmNI supports dynamic contrast construction and provides interactive visualizations (e.g. volcano plots and PCA). These features enable iterative, hypothesis-driven analysis.

OmNI’s limma-powered models excel in various scenarios common in omics research. For instance, researchers can readily:

- Analyze time-series experiments: Investigate how molecular profiles change over multiple time points and identify dynamic responses.
- Deconvolute effects in multi-factor designs: Simultaneously assess the impact of multiple experimental variables (e.g. drug treatment, genotype, and sex) and their potential interactions.
- Account for batch effects and confounding variables: As demonstrated in our case study, limma’s ability to model covariates and utilize duplicateCorrelation (or other appropriate methods for dependent samples) effectively isolates true biological variation from technical noise, ensuring reliable conclusions. This is particularly crucial for large-scale studies where samples are processed across different days, operators, or reagent lots.
- Perform paired analyses: Compare samples from the same individual under different conditions (e.g. pre- and post-treatment), increasing statistical power by removing inter-individual variability.

In our fentanyl case study, we specified a formula-based fixed-effects linear model to estimate both global and strain-specific treatment effects. To account for technical variability, the dissection session (batch) was explicitly modeled as a covariate, and batch-related variation was rigorously handled using duplicateCorrelation. This approach is crucial because it ensures that the variance attributable to batch is properly accounted for within the model, rather than being removed beforehand, a practice that could inadvertently obscure true biological variation. Following this modeling, PCA visualization corrected for batch effects (Fig. 3b) revealed a partial separation of treatment groups, directly confirming the underlying biological structure identified by our model. Furthermore, empirical Bayes moderation (eBayes with robust = TRUE) was applied to stabilize variance estimates across thousands of features. This significantly enhances statistical power and interpretability, especially beneficial in small-sample conditions. OmNI automatically generated and validated a complete design matrix (Fig. 3c), a critical step for preventing collinearity and ensuring sound statistical inference.

OmNI’s interactive outputs facilitate deep, user-driven exploration. For instance, selecting a feature like Siah2 directly from a volcano plot triggers a Glimma-powered dashboard (Fig. 3d). This dashboard instantly displays its log_2_ fold change, sample-level intensities, and UniProt annotation, allowing for a detailed, interactive inspection of individual feature behavior. To facilitate mechanistic understanding and biological interpretability, OmNI seamlessly integrates gene set enrichment analysis (GSEA) directly into its modeling workflow. Features are ranked by logFC and *P*-value, then passed to clusterProfiler for comprehensive pathway enrichment analysis across numerous curated databases, including Reactome, KEGG, and GO, which users can select at will using a dropdown menu. For example, a dot plot of the top 20 enriched Reactome pathways revealed significant associations with transcriptional regulation, RNA processing, and mitochondrial function (Fig. 3e). These findings are consistent with the identities of the global differentially expressed proteins (DEPs) and phosphoproteins (DEPhos) identified in our study. In parallel, OmNI enables network-based visualization of enriched GO terms using clusterProfiler’s “cnetplot” functionality. This approach groups enriched biological processes based on shared gene membership, effectively revealing functional modules such as chromatin remodeling, metabolic regulation, and cytoskeletal organization (Fig. 3f). These context-aware visualizations are invaluable for bridging the gap between statistical output and mechanistic insight, guiding interpretation even when the number of differentially expressed features is modest.

Together, this illustrates OmNI’s ability to manage complex linear modeling workflows with statistical rigor and transparency. Even when traditional differential testing yields a limited number of features due to data sparsity or high variance, OmNI provides a solid analytical scaffold for signal extraction and hypothesis refinement. These global model outputs laid the foundation for downstream integration using the *S*-score method, which enhanced sensitivity by combining signals across omic layers.

### Covariate-adjusted multi-omic integration using an enhanced *S*-score framework

In multi-omics studies, researchers integrate data across layers such as genomics, transcriptomics, and proteomics using either early integration (combining data before analysis) or late integration (combining results post-analysis) [19]. The *S*-score is an early integration method that consolidates multi-omic data into a weighted, normalized gene-level score. By capturing cross-omic interactions and reducing layer-specific noise, it boosts statistical power and enhances detection of biologically meaningful signals often missed by single-omics approaches.

OmNI extends the original *S*-score framework by integrating it with limma-powered linear modeling, enabling covariate-adjusted multi-omic integration while preserving biological interpretability (Fig. 4a). Unlike traditional *S*-score approaches that operate on raw log-fold changes (i.e. mean group differences), OmNI synthesizes model-derived logFCs that account for experimental complexity—including batch effects, technical replicates, and interaction terms. In the fentanyl study, e.g., logFCs were adjusted for dissection session and strain-specific variation, ensuring technical confounders were considered before integration. This prevents latent biases from propagating into downstream prioritization, a major limitation of naive data fusion strategies.

Another key innovation in OmNI’s implementation is its customizable weighting scheme, which enables users to tailor integration based on biological context (Fig. 4a). While the original *S*-score assigns fixed weights based on dataset size (1/√n), OmNI allows additional dynamic user-defined weighting. For example, transcriptomic data can be down-weighted relative to proteomic or phosphoproteomic layers when the focus is on post-transcriptional or signaling-level regulation. OmNI’s weighting system empowers domain-specific prioritization, but adjustments should be guided by strict biological rationale. Equal weighting is appropriate (default in OmNI) for balanced discovery across layers, whereas biased weighting—for instance, emphasizing phosphoproteomics in signaling studies—can enhance specificity but requires careful evaluation to avoid misrepresentation.

OmNI successfully identified coordinated fentanyl-responsive features (Fig. 4b). Among the 81 significantly regulated features revealed via *S*-score integration (Fig. 4c), the consistent downregulation of Siah2 (*S*-score = −26.68, adjusted *P*-value = 1.29e−157) across all strains emerged as a novel facet of the acute opioid response. Siah2, a key ubiquitin-proteasome regulator involved in hypoxia adaptation via PHD–HIF-1α signaling, may be repressed as a neuroprotective response to fentanyl induced hypoxic stress, enhancing oxygen-sensitive pathways and modulating intracellular stress signaling [20]. Its suppression also implicates Siah2 in opioid-driven neuroplasticity, potentially altering protein turnover, synaptic stability, and early molecular events in tolerance development [21]. The significant repression of Siah2 was part of a much broader, multi-faceted molecular response to fentanyl that OmNI’s integrative framework successfully captured. The findings point to a major reprogramming of gene expression and protein synthesis, evidenced by the concurrent upregulation of factors controlling chromatin structure (H2az2), transcription termination (Ttf2), RNA processing (Rbm48), and ribosome production (Fcf1), while an RNA methyltransferase (Mettl15) was suppressed [22]. This rewiring of the cellular machinery occurred alongside clear indicators of emerging neuroplasticity and stress. Proteins involved in stress adaptation (Nupr1) and neuronal processes (Bend7) were downregulated, while cytoskeletal components essential for neuronal architecture (Myh8) were upregulated. Furthermore, OmNI detected a potential calming of innate immune pathways through the downregulation of the antiviral protein Mx1 [23]. The observed changes paint a picture of early neuroplastic adaptation driven by a deep remodeling of the gene expression landscape, offering novel insights into fentanyl’s complex neurobiological impact.

For the strain-specific response, OmNI identified 501 significantly differential features, spanning 443 unique genes (Fig. 4d). The extent of this molecular response varied considerably across strains, with 129S1/SvImJ (129) exhibiting the most pronounced changes (accounting for 12.5% of differential features), while NZO/HlLtJ (NZO) displayed the weakest response (9.9%) (Fig. 4e).

Focusing on the A/J and CAST/EiJ strains, which exhibit opposing sensitivities to opioids, we used OmNI to map their distinct molecular responses to fentanyl. Our analysis first identified a convergent core response program, suggesting shared adaptive mechanisms. This included common alterations in protein homeostasis and stress response, marked by the regulation of factors involved in ubiquitination (Siah2 and Fbxl2) and cellular stress (Xaf1). Both strains also displayed convergent shifts in gene expression regulation (Med13 and Rbm48) and neuronal infrastructure, including cytoskeletal remodeling (Myh8), extracellular matrix changes (Ecm1), and the modulation of cholinergic neurotransmission (Chrna4-S468/S470) [24]. However, OmNI also uncovered striking divergent molecular signatures that likely underpin their different phenotypes. The less-sensitive A/J strain showed a unique upregulation of pathways related to cellular metabolism and transport, including key enzymes and transporters for nutrients and neurotransmitter precursors (Aldh8a1, Tmlhe, and Slc38a2), which were downregulated in the sensitive CAST/EiJ strain. Conversely, a suite of proteins involved in neuro-immune signaling and neuronal development (C8a, Bmp2, and Zc3hav1) were suppressed in A/J but upregulated in CAST/EiJ [23]. These marked, opposing divergences in metabolic, structural, and immune pathways highlight how distinct genetic architectures can shape profoundly different molecular trajectories in response to fentanyl.

These findings firmly establish OmNI as a next-generation platform for precision multi-omics, combining limma’s statistical power with *S*-score synthesis. Its covariate-aware, cross-layer integration reveals hidden biological patterns. In acute fentanyl exposure, OmNI uncovered a novel neuroadaptive state involving altered proteostasis, transcriptional reprogramming, and neuronal signaling. This coordinated multi-omic response, undetectable in single datasets, offers a new lens into how opioids reshape brain function. These insights provide targets for exploring acute opioid action, tolerance, dependence, and therapeutic intervention.

### PCSF network analysis identifies conserved and strain-specific fentanyl responsive modules

OmNI’s network analysis framework applies the PCSF algorithm to integrate multi-omics *S*-scores with a curated interaction network, enabling the discovery of biologically robust and mechanistically interpretable subnetworks (Fig. 5a). By using the integrated *S*-scores as node prizes, this approach moves beyond simple pathway lists to identify the key regulatory hubs and functional modules that are most perturbed by fentanyl exposure.

In the global, pan-strain analysis of the fentanyl response, the PCSF network was organized around the central hub MTUS2, a key microtubule-associated protein involved in scaffolding and cellular signaling. Other prominent nodes include CAMK2A (calcium/calmodulin-dependent protein kinase II alpha), DCDC2B (doublecortin domain containing 2B), ZFYVE21 (zinc finger FYVE-type containing 21), and ZNF136 (zinc finger protein 136). The network was most significantly enriched for pathways related to the “epigenetic regulation of gene expression and chromatin organization” (FDR = 0.045), including the key epigenetic factors EPOP (EPO receptor binding protein), EZH1 (enhancer of zeste homolog 1, a histone methyltransferase of the PRC2 complex), SETD1A (SET domain containing 1A, a histone H3K4 methyltransferase), and SETDB2 (SET domain bifurcated 2, another histone methyltransferase) (Fig. 5b). This finding suggests that the conserved, core response to acute fentanyl exposure across all strains involves a significant reprogramming of the transcriptional and epigenetic landscape, anchored by a central structural and signaling hub.

While the global network revealed a shared theme of gene expression regulation, strain-specific network analysis uncovered starkly divergent molecular strategies between the opioid-sensitive CAST/EiJ strain and the less-sensitive A/J strain. These differences in network topology provide a powerful mechanistic hypothesis for their opposing behavioral phenotypes. The network of the less-sensitive A/J strain was organized around hubs deeply involved in metabolism and neurotransmitter transport (Fig. 5c and Supplementary Fig. S3a and b). The top influential hub was SLC38A2 (solute carrier family 38 member 2), a critical amino acid transporter, with other prominent nodes including TMBIM6 (transmembrane BAX inhibitor motif containing 6, a transmembrane protein involved in stress response and apoptosis regulation), ALDH3A1 (aldehyde dehydrogenase 3 family member A1, an aldehyde dehydrogenase involved in detoxification), and SLC6A4 (solute carrier family 6 member 4, the serotonin transporter). This suggests that the molecular response in the resistant A/J strain is geared toward managing metabolic stress and maintaining control over neurotransmitter homeostasis.

In contrast, the network of the highly sensitive CAST/EiJ strain was centered on pathways of programmed cell death (apoptosis) and protein degradation (Fig. 5d). The top influential hub was CASP9 (Caspase-9), the primary initiator-caspase of the intrinsic apoptotic pathway. This pro-death signal was reinforced by other prominent hubs, including TSPAN3 (tetraspanin 3) and the E3 ubiquitin ligases RNF32 (ring finger protein 32) and RNF149 (ring finger protein 149). This architecture suggests that in the sensitive strain, the acute fentanyl exposure triggers a severe cellular stress response that activates protein turnover and programmed cell death pathways, a molecular signature that is absent in the resistant strain.

In summary, by embedding covariate-adjusted *S*-scores within an interaction topology, OmNI’s PCSF analysis successfully translates complex omics data into actionable biological hypotheses. It identified a conserved core response to fentanyl centered on gene expression, but more importantly, it resolved functionally distinct, strain-specific subnetworks. The divergence between a metabolic/transport-oriented response in the resistant A/J strain and an apoptosis-driven response in the sensitive CAST/EiJ strain provides a novel, system-level mechanism to explain their differential sensitivity to opioids.

### OmNI’s comprehensive reporting facilitates reproducibility and collaboration

**Figure 6.**
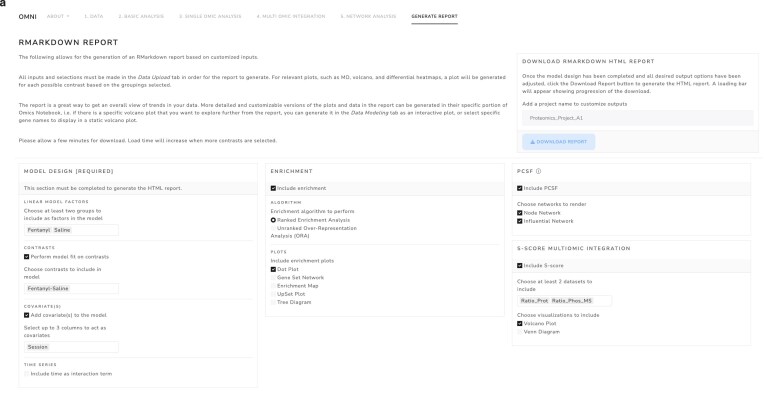
Comprehensive HTML summary report generation screenshot of the interactive report generation interface, which allows for selections of data processing preferences as well as inclusion or exclusion of additional analyses such as enrichment, multi-omic integration, and PCSF network analysis based on user case.

OmNI’s automated report generation module compiles multi-omic analyses into a unified, interactive HTML document, promoting reproducibility and collaboration (Fig. 6 and Supplementary Data 1). It features interactive visualizations (Plotly volcano plots and Glimma dashboards), downloadable tables (CSV/TSV), and publication-ready figures (PDF/SVG) with statistical summaries (adjusted *P*-values, effect sizes, variance metrics). Network outputs exported as Cytoscape-compatible files (XGMML, SIF) for subnetwork refinement or merging with external data. The module’s dynamic HTML report allows interactive exploration via widgets toggling analytical states (e.g. pre-/post-normalization PCA plots) or filtering heatmaps by significance thresholds. Users can zoom into network modules to inspect node-edge relationships or adjust volcano plot cutoffs. Analytical provenance is maintained with embedded R code and session metadata (R version, dependencies, parameters), ensuring transparency and replicability. Outputs are structured in nested folders by analysis type (e.g. DE_Results and Network_Modules), containing the various outputs. README files provide format details, reuse guidelines, and version info, minimizing curation and enhancing focus on biological insights.

For the fentanyl study, OmNI efficiently generated a comprehensive interactive HTML report within 15 min, with fully integrated interactive networks, detailed data tables, and 40+ publication-ready diagrams. By seamlessly integrating interactive visual exploration, exportable publication-quality outputs, and analytical transparency, OmNI’s reporting module significantly streamlines the workflow from raw data to biological insight. Its implementation with open-source platforms such as R and Cytoscape facilitates easy deployment, while its modular framework ensures adaptability to emerging omics technologies, including single-cell and spatial omics. This emphasis on reproducibility and accessibility democratizes advanced multi-omics analysis, fostering collaboration across computational and experimental research communities.

### Comparison with other tools

**Figure 7.**
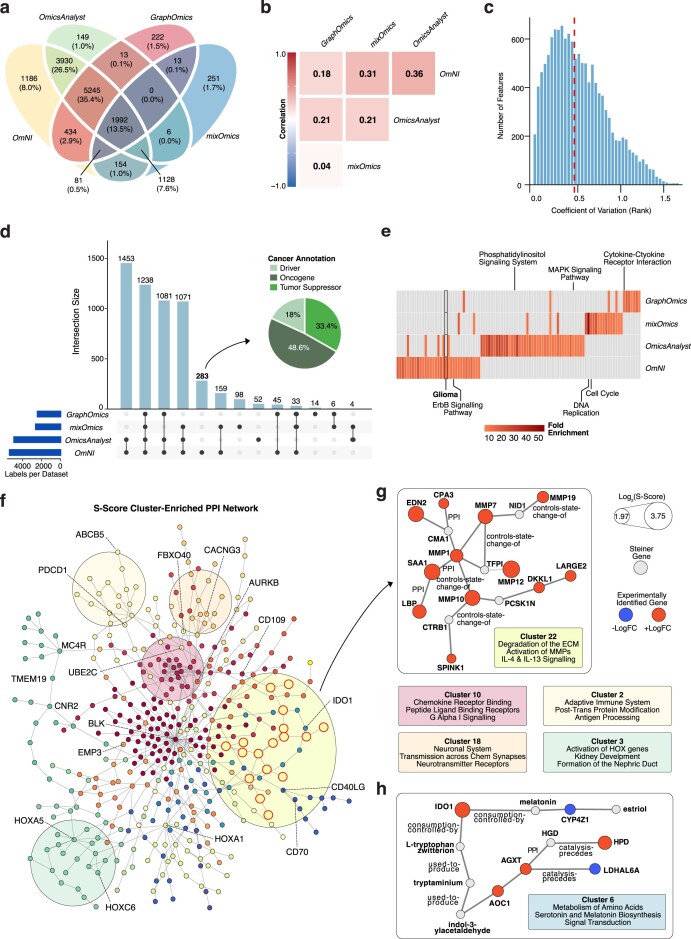
Comparison of multi-omic integration tools. (**a**) Venn diagram of features identified as significantly altered (see the “Materials and methods” section) by OmNI, OmicsAnalyst, GraphOmics, and mixOmics. (**b**) Correlation heatmap showing the rank correlation of significant features identified by the four tools. (**c**) Histogram of the coefficient of variation (CV) for all ranked features. (**d**) UpSet plot comparing the overlap of significantly altered features overlapped with the CancerMine database detailing the Cancer Annotation breakdown. The pie chart shows the features identified uniquely by OmNI that are known key players in oncogenesis. (**e**) Heatmap illustrates enriched biological terms derived from the top 100 ranked features identified by each analytical tool. Pathways of particular relevance to GBM are annotated. (**f**) OmNI-specific protein–protein interaction (PPI) network highlighting distinct functional clusters identified through enrichment analysis. (**g**) Representative PPI subnetwork as well as highlighted cluster enrichment associated with extracellular matrix remodeling, immune signaling, and neuronal processes. Nodes are colored by gene expression fold change, and selected significantly enriched biological pathways (adjusted *P* < .05) are indicated. (**h**) PPI subnetwork corresponding to Cluster 6, representing an integrated metabolite–gene module enriched for amino acid metabolism and neurotransmitter biosynthesis pathways.

To benchmark OmNI’s performance, we compared it against several established and highly cited multi-omics integration tools, reflecting their widespread use in the community: OmicsAnalyst [25], GraphOmics [26], and mixOmics [27]. We analyzed a CPTAC glioblastoma (GBM) dataset [28] using all four frameworks. The comparative analysis, summarized in Fig. 7, demonstrates OmNI’s superior sensitivity, feature stability, and unique capacity for system-level biological interpretation.

A comparison of significantly altered features using a Venn diagram (Fig. 7a) reveals both broad consensus and tool-specific discoveries. Notably, OmNI identifies a significant set of unique features (*n* = 1186) not captured by the other frameworks, while also sharing a large core of features (*n* = 5245) with all other tools.

We next examined feature prioritization and rank stability. A heatmap of pairwise rank correlations (Fig. 7b) revealed significant methodological divergence, as evidenced by generally low Spearman correlation values, such as between OmNI and OmicsAnalyst (*ρ *= 0.36) and OmNI and mixOmics (*ρ *= 0.31). This reflects that each tool prioritizes features differently based on its underlying integration strategy. Next, an analysis of feature rank stability using the coefficient of variation (CV) (Fig. 7c) confirmed that while a core set of features represented a stable consensus (low CV), many features exhibited rank variability across tools, as indicated by the median CV. OmNI’s approach proves effective, as it successfully identifies both these robust, cross-platform consensus biomarkers (low CV features) and potentially novel discoveries (high CV features).

To further dissect the feature overlap, an UpSet plot (Fig. 7d) was generated. This analysis confirmed a large intersection between OmNI and OmicsAnalyst (*n* = 1453) but also highlighted a distinct set of 283 features identified exclusively by OmNI. A cancer-focused annotation of this unique set (Fig. 7d, pie chart) shows they are highly relevant, with over 51% being established oncogenes or tumor suppressors. This demonstrates OmNI’s sensitivity in capturing critical, cancer-driving features missed by other methods.

Despite the divergence in feature ranking, OmNI’s prioritization proved the most biologically coherent. An enrichment analysis on the top 100 ranked features (Fig. 7e) shows that while both OmNI and OmicsAnalyst’s results are strongly enriched for key cancer processes like “PI3K signaling” and “MAPK,” OmNI’s list is uniquely enriched for pathways highly specific to GBM, such as “Glioma” and “ErbB Signalling Pathway.” This superior, disease-specific enrichment visually demonstrates that OmNI’s *S*-score ranking provides a more comprehensive and clinically relevant biological profile.

Beyond feature lists, OmNI’s strength lies in its ability to structure these findings into actionable biological context—a step not all tools, like mixOmics, provide natively. OmNI’s integrated network (Fig. 7f) is dense and multi-layered, producing a more cohesive representation of tumor biology. This network view allows for the exploration of specific functional modules, such as a subnetwork associated with extracellular matrix remodeling and immune signaling (Fig. 7g). Crucially, OmNI was uniquely able to construct integrated, multi-omic modules, as exemplified by Cluster 6 (Fig. 7h), which represents a true gene-metabolite module linking amino acid metabolism with neurotransmitter biosynthesis pathways. This ability to move from high-level feature identification to integrated, system-level network insights highlights OmNI as a comprehensive and powerful tool for multi-omics discovery.

A complete, interactive HTML report detailing the full results of this CPTAC GBM analysis is available as Supplementary Data 2.

#### Performance and usability

The scalability of a bioinformatics tool is critical for handling the ever-increasing size of multi-omics datasets. Table 2 summarizes the core capabilities of OmNI and the other benchmark tools. This table highlights OmNI’s unique combination of features, including its support for a wider range of omics data types, its interactive GUI, and its inclusion of key network algorithms, making it a powerful and versatile end-to-end solution.

**Table 1.** Tool feature comparison

Feature	OmNI	mixOmics	OmicsAnalyst 2.0	GraphOmics
Platform	Interactive GUI	R Package (Script-based)	Web page	Web page
Supported omics types	All 5 omics layers	Multiple (Flexible)	Proteomics, transcriptomics, metabolomics	Proteomics, transcriptomics, metabolomics
Integrated output	*S*-score	Latent variables (DIABLO, PLS-DA)	Composite scores	Multi-omic factor analysis
Network algorithms	Yes (PCSF)	No	Yes (Limited)	Yes (Limited)
Visualization	High-quality, interactive	Plotly (in R)	Plotly (in UI)	Plotly (in UI)
Workflow	End-to-end	Requires manual steps (preprocessing)	Fragmented (UI Issues)	Fragmented (Slow)
Runtime and memory usage	30–45 min and 3.5 GB	4–5 h	30–45 min	1+ h
User-defined parameters and interventions	Few required, many available	Many required	Some required, many available	Few required, few available

**Table 2.** Software package overview

Package		Version	Link	Use
UI				
	Bsicons	0.1.2	CRAN	Bootstrap icons in Shiny app UI
	Bslib	0.8.0	CRAN	Shiny UI CSS styling
	DT	0.33	CRAN	Data table formatting and styling
	GeneTonic	2.8.0	BioConductor	Data table color formatting
	htmltools	0.5.8.1	CRAN	HTML outputs
	kableExtra	1.4.0	CRAN	Table formatting
	markdown	1.13	CRAN	Report generation
	rmarkdown	2.28	CRAN	Report generation
	shiny	1.9.1	CRAN	Interactive tool base
	shinycssloaders	1.1.0	CRAN	Loading animations
Data and package load/clean				
	Biobase	2.64.0	BioConductor	Load base BioConductor functions
	BiocManager	1.30.25	CRAN	Bioconductor package management
	devtools	2.4.5	CRAN	Figure formatting and saving
	openxlsx	4.2.7	CRAN	Excel file formatting and downloads
	pacman	0.5.1	CRAN	Package management and loading
	readr	2.1.5	CRAN	File reading and formatting
	tidyverse	2.0.0	CRAN	Data formatting and manipulation
Normalization				
	NormalyzerDE	1.22.0	BioConductor	MedianMAD normalization
	vsn	3.72.0	BioConductor	VSN normalization
Gene/protein ID mapping				
	EnsDb.Hsapiens.v86	2.99.0	BioConductor	Human Ensembl database annotations
	EnsDb.Mmusculus.v79	2.99.0	BioConductor	Mouse Ensembl database annotations
	org.Ce.eg.db	3.19.1	BioConductor	Worm gene ID annotation
	org.Dm.eg.db	3.19.1	BioConductor	Fly gene ID annotation
	org.Dr.eg.db	3.19.1	BioConductor	Zebrafish gene ID annotation
	org.Hs.eg.db	3.19.1	BioConductor	Human gene ID annotation
	org.Mm.eg.db	3.19.1	BioConductor	Mouse gene ID annotation
	org.Rn.eg.db	3.19.1	BioConductor	Rat gene ID annotation
	org.Sc.sgd.db	3.19.1	BioConductor	Yeast gene ID annotation
Statistical analysis				
	limma	3.60.4	BioConductor	Differential expression analysis
	pcaMethods	1.96.0	CRAN	PCA calculations
	psych	2.4.6.26	CRAN	Generate statistical summary table
	uwot	0.2.2	CRAN	Generate UMAP
Plotting				
	colourpicker	1.3.0	CRAN	Shiny color picker UI
	colourvalues	0.3.9	CRAN	Maps viridis color palette
	ComplexHeatmap	2.20.0	BioConductor	Customizable heatmap generation
	cowplot	1.1.3	CRAN	Arrange multiple plots together
	ggcorrplot	0.1.4.1	CRAN	ggplot2-based correlation plot
	ggplot2	3.5.1	CRAN	Base plotting function
	ggfortify	0.4.17	CRAN	ggplot2-based stat plotting tools
	ggpubr	0.6.0	CRAN	ggplot2-based QQ plots
	ggrepel	0.9.6	CRAN	ggplot2-based labels within plots
	ggridges	0.5.6	CRAN	ggplot2-based ridgeline plots
	ggupset	0.4.0	CRAN	ggplot2-based UpSet plots
	ggvenn	0.1.10	CRAN	ggplot2-based Venn diagrams
	Glimma	2.14.0	BioConductor	Interactive limma volcano plot output
	heatmaply	1.5.0	CRAN	Interactive heatmaps
	pheatmap	1.0.12	CRAN	Corerlation heatmaps
	plotly	4.10.4	CRAN	Interactive ggplot2 objects
	VIM	6.2.2	CRAN	Missing value visualization
Enrichment				
	clusterProfiler	4.12.6	BioConductor	Enrichment calculation and visualization
	enrichplot	1.24.2	BioConductor	Addtl enrichment visualization
	KEGGREST	1.44.1	BioConductor	KEGG pathway ID for visualization
	pathview	1.44.0	BioConductor	KEGG pathway visualization
PCSF network visualization				
	igraph	2.0.3	CRAN	Network graph object creation
	influential	2.2.9	CRAN	Calculation of influential nodes
	IOR-Bioinformatics/PCSF	0.99.1	GitHub	Prize-collecting Steiner forest algorithm application for networks
	visNetwork	2.1.2	CRAN	Interactive network visualization

The runtime and memory usage compares the total runtime and peak memory usage for each tool during network analysis on the CPTAC dataset. OmNI completed the analysis in <1 h with a peak memory usage of 3.5 GB. This demonstrates a significant advantage in efficiency, particularly when compared to mixOmics, which took a few hours to run on this data, and GraphOmics, which was notably slow. This efficiency is a core benefit, as it reduces computational overhead and allows researchers to perform iterative analyses more quickly.

For our original fentanyl study, OmNI efficiently generated a comprehensive interactive HTML report within 15 min, with fully integrated interactive networks, detailed data tables, and 40+ publication-ready diagrams.

## Discussion

OmNI represents a crucial advancement in the burgeoning field of multi-omics. Developed as a user-friendly, open-source platform, OmNI uniquely addresses the critical gap left by existing tools that often narrow their focus to single data types or offer limited, non-interactive integration. By robustly and cohesively integrating transcriptomic, proteomic, phosphoproteomic, and metabolomic datasets within a single modular framework, OmNI offers researchers an accessible, powerful, and scalable solution for navigating the complexity of biological systems.

The persistent opioid crisis, profoundly impacted by fentanyl’s potency, highlights the urgent need for system-level analyses that can unravel the intricate molecular interactions governing drug response. Leveraging OmNI, our analysis of deep proteomic and phosphoproteomic data from the CCF panel exposed to fentanyl unequivocally demonstrated its effectiveness as a versatile analytical platform. OmNI’s integrative approach uniquely illuminated fentanyl induced molecular changes, revealing coordinated responses across signaling pathways, protein interaction networks, and metabolic processes that were otherwise undetectable in single-layer analyses. Specifically, we uncovered a novel neuroadaptive state characterized by the convergent downregulation of proteins involved in ubiquitination, stress response (e.g. Siah2 and Nupr1), and extracellular matrix remodeling (e.g. Ecm1 and Bend7), alongside upregulation of factors influencing RNA processing (e.g. Rbm48), chromatin structure (e.g. H2az2), and cholinergic neurotransmission (e.g. Chrna4 phosphorylation). These findings underscore OmNI’s capability to resolve novel, biologically meaningful signals and molecular targets that are critical for understanding the system-level impact of opioids on brain function.

A core strength of OmNI lies in its context-aware interactivity, fostering a dynamic interplay between exploratory and hypothesis-driven analysis. Users can directly explore enriched pathways, dynamically linking them to relevant molecular features within interactive visualizations. This unique integration markedly accelerates biological interpretation and hypothesis generation. Furthermore, OmNI’s modular architecture, built on open-source R Markdown and Cytoscape, ensures exceptional scalability and maintainability, facilitating seamless adoption across diverse computational infrastructures, from local workstations to high-performance computing environments. Its capacity to illuminate strain-specific molecular responses, as evidenced by our A/J and CAST/EiJ analyses, positions OmNI as an invaluable tool for precision systems genetics.

While OmNI significantly advances multi-omics analysis, integrating large-scale, heterogeneous datasets inherently presents computational demands that scale with data complexity, necessitating appropriate high-performance resources for optimal performance. Moreover, effective multi-omic integration demands meticulous experimental design and careful upstream normalization strategies to mitigate biases. Continuous enhancements to OmNI’s computational efficiency, user interface, and analytical modules—including the integration of additional multi-omics approaches such as mixOmics [27] and MOFA [29]—supported by collaborative efforts across computational and biological research communities, will further address these considerations and expand its utility.

In summary, OmNI represents a significant step in the analytical landscape of multi-omics research. By enabling sophisticated, user-driven integration of diverse biological datasets and delivering intuitive, interactive outputs, it facilitates unprecedented insights into complex phenomena such as opioid responses. This integrative capability positions OmNI as an indispensable tool for systems biology, advancing both fundamental research and the identification of novel therapeutic targets relevant to critical public health challenges.

## Supplementary Material

lqaf206_Supplemental_Files

## Data Availability

The mass spectrometry proteomics data have been deposited to the ProteomeXchange Consortium via the MassIVE partner repository with the dataset identifier MSV000098604. OmNI is freely available for download from https://github.com/gracerhpotter/OmNI and https://doi.org/10.5281/zenodo.17714506, and is also accessible via a web interface at https://emili-laboratory.shinyapps.io/omni/.
