# Supplementary material for: OmNI: a modular open-source framework for interactive multi-omics data integration and visualization
Source: NAR Genom Bioinform. 2026 Jan 10;8(1):lqaf206. doi: 10.1093/nargab/lqaf206 (PMC12789801; doi:10.1093/nargab/lqaf206)
Supplement: lqaf206_Supplemental_Files [file lqaf206_supplemental_files.zip › Supplementary_Figures.pdf]

Supplementary Fig. 1

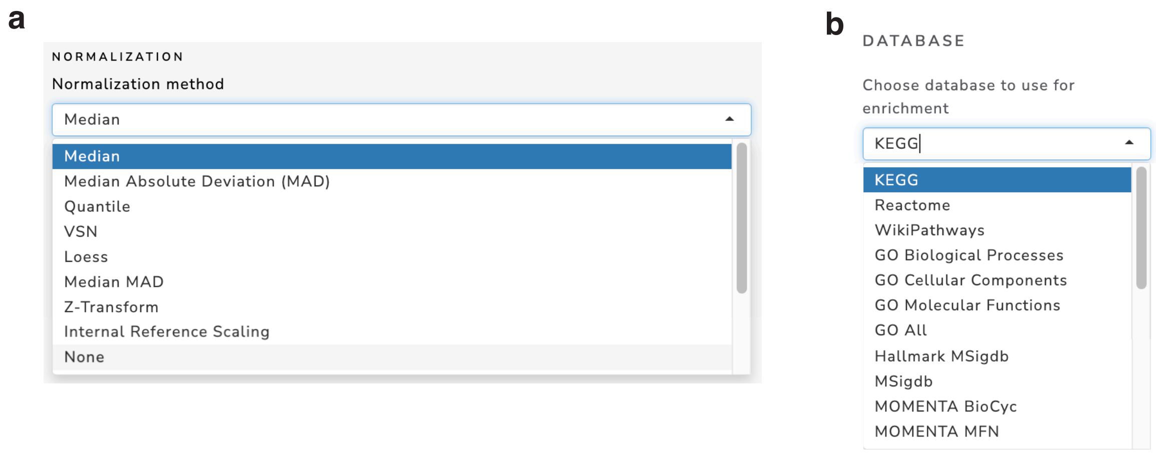

Supplementary Fig. 2

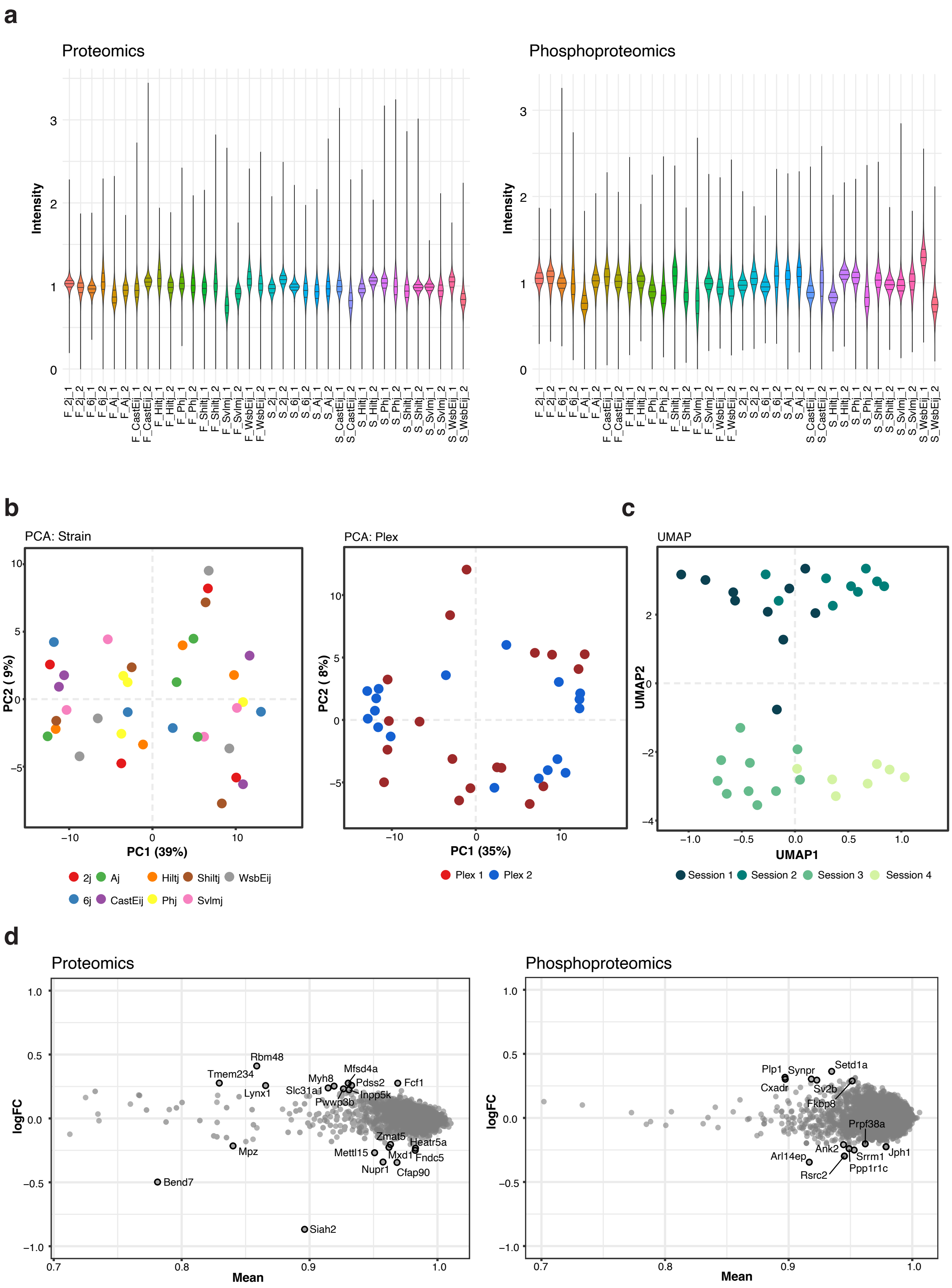

Supplementary Fig. 3

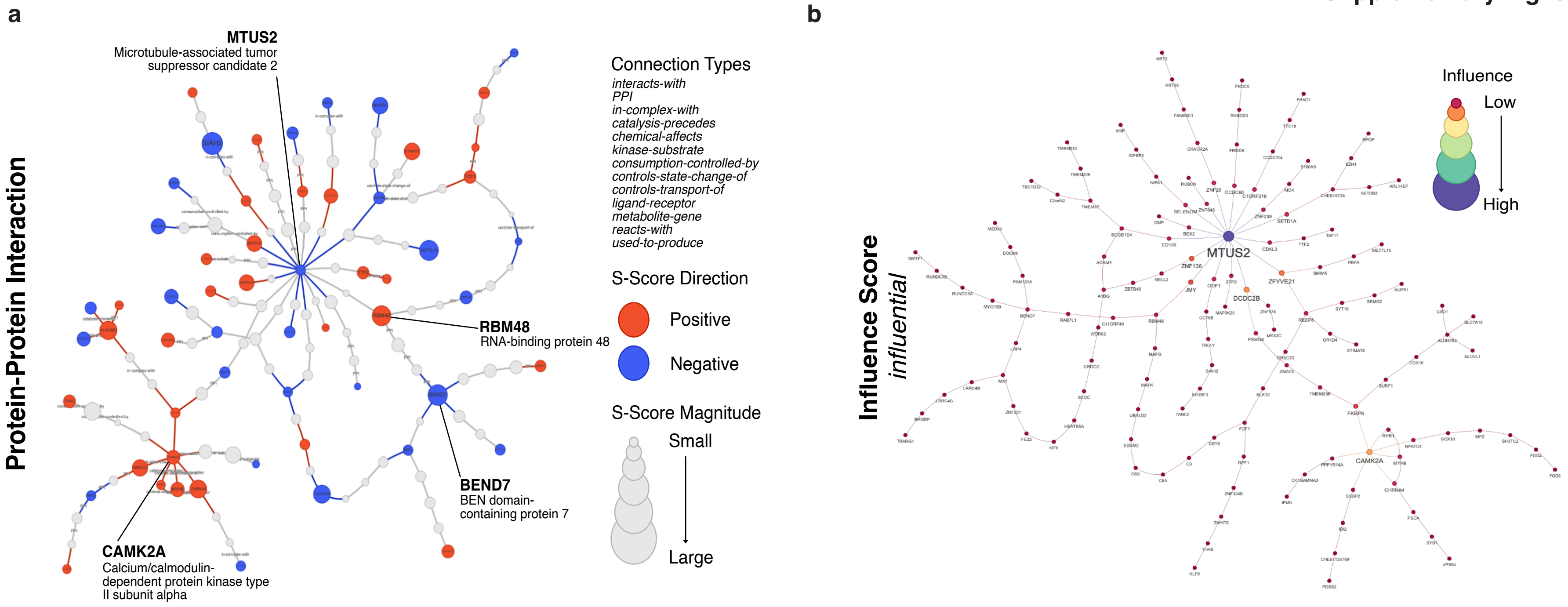

Supplementary Figure Legends

Supplementary Fig. 1: Enrichment and normalization interface

a. Screenshot of OmNI user interface dropdown selection of normalization options.

b. Screenshot of OmNI user interface dropdown selection of pathway enrichment database options. Options vary slightly based on species selection; species like human and mouse have more available databases to choose from.

Supplementary Fig. 2: Continued evaluation and data quality assessment of datasets

a. Violin plots showing raw data distribution for both proteomics (left) and phosphoproteomics (right) data across all samples.

b. PCA plots for phosphoproteomics data colored by covariates "Strain" (left) and "Plex" (right) showing weak grouping.

c. UMAP plot for phosphoproteomics data colored by "Session".

d. Mean Difference (MD) plots of Fentanyl vs. Saline overall comparison for both proteomics (left) and phosphoproteomics (right) datasets with a few features of largest magnitude logFC labeled.

Supplementary Fig. 3: Protein-Protein interaction and influence networks

a. Protein-Protein Interaction (PPI) network for Fentanyl vs. Saline comparison across all strains, highlighting a few central proteins.

b. Influential protein network for Fentanyl vs. Saline comparison across all strains showing protein influence within the overall PPI structure, highlighting MTUS2 as a central interaction hub.
