# Supplementary material for: OmNI: a modular open-source framework for interactive multi-omics data integration and visualization
Source: NAR Genom Bioinform. 2026 Jan 10;8(1):lqaf206. doi: 10.1093/nargab/lqaf206 (PMC12789801; doi:10.1093/nargab/lqaf206)
Supplement: lqaf206_Supplemental_Files [file lqaf206_supplemental_files.zip › CPTAC_GBM_OmNI_Report.html.zip!/CPTAC_GBM_OmNI_Report.html]

OmNI Report


# OmNI Report

#### September 11, 2025

## Image Folder

```
## [1] "~/Downloads/CPTAC_GBM_OmNI_Report_2509111619"
```

> Right-click on any images to open them in a new tab, save them to
> your computer, or copy them.

# Dataset Information

An overview of the dataset uploaded and groups, contrasts, and
expression set used in generating the analyses in the report.

## Annotation File

| SampleName | prot | phospho | met | rna | Group | Subtype |
| --- | --- | --- | --- | --- | --- | --- |
| C3L\_00104 | C3L-00104 | C3L-00104 | NA | C3L-00104 | Tumor | Proneural |
| C3L\_00365 | C3L-00365 | C3L-00365 | C3L-00365 | C3L-00365 | Tumor | Classical |
| C3L\_00674 | C3L-00674 | C3L-00674 | C3L-00674 | C3L-00674 | Tumor | Mesenchymal |
| C3L\_00677 | C3L-00677 | C3L-00677 | C3L-00677 | C3L-00677 | Tumor | Proneural |
| C3L\_01040 | C3L-01040 | C3L-01040 | NA | C3L-01040 | Tumor | Classical |
| C3L\_01043 | C3L-01043 | C3L-01043 | C3L-01043 | C3L-01043 | Tumor | Mesenchymal |
| C3L\_01045 | C3L-01045 | C3L-01045 | C3L-01045 | C3L-01045 | Tumor | Mesenchymal |
| C3L\_01046 | C3L-01046 | C3L-01046 | NA | C3L-01046 | Tumor | Mesenchymal |
| C3L\_01048 | C3L-01048 | C3L-01048 | C3L-01048 | C3L-01048 | Tumor | Classical |
| C3L\_01049 | C3L-01049 | C3L-01049 | C3L-01049 | C3L-01049 | Tumor | Mesenchymal |
| C3L\_01061 | C3L-01061 | C3L-01061 | NA | C3L-01061 | Tumor | Classical |
| C3L\_01142 | C3L-01142 | C3L-01142 | NA | C3L-01142 | Tumor | Mesenchymal |
| C3L\_01146 | C3L-01146 | C3L-01146 | NA | C3L-01146 | Tumor | Proneural |
| C3L\_01149 | C3L-01149 | C3L-01149 | C3L-01149 | C3L-01149 | Tumor | Proneural |
| C3L\_01154 | C3L-01154 | C3L-01154 | NA | C3L-01154 | Tumor | Classical |
| C3L\_01155 | C3L-01155 | C3L-01155 | NA | C3L-01155 | Tumor | Classical |
| C3L\_01156 | C3L-01156 | C3L-01156 | NA | C3L-01156 | Tumor | Classical |
| C3L\_01157 | C3L-01157 | C3L-01157 | C3L-01157 | C3L-01157 | Tumor | Proneural |
| C3L\_01327 | C3L-01327 | C3L-01327 | NA | C3L-01327 | Tumor | Classical |
| C3L\_01834 | C3L-01834 | C3L-01834 | NA | C3L-01834 | Tumor | Proneural |
| C3L\_01887 | C3L-01887 | C3L-01887 | C3L-01887 | C3L-01887 | Tumor | Classical |
| C3L\_02041 | C3L-02041 | C3L-02041 | C3L-02041 | C3L-02041 | Tumor | IDH mutant |
| C3L\_02465 | C3L-02465 | C3L-02465 | C3L-02465 | C3L-02465 | Tumor | Mesenchymal |
| C3L\_02504 | C3L-02504 | C3L-02504 | C3L-02504 | C3L-02504 | Tumor | Proneural |
| C3L\_02542 | C3L-02542 | C3L-02542 | C3L-02542 | C3L-02542 | Tumor | Proneural |
| C3L\_02642 | C3L-02642 | C3L-02642 | C3L-02642 | C3L-02642 | Tumor | Classical |
| C3L\_02704 | C3L-02704 | C3L-02704 | C3L-02704 | C3L-02704 | Tumor | Mesenchymal |
| C3L\_02705 | C3L-02705 | C3L-02705 | C3L-02705 | C3L-02705 | Tumor | Mesenchymal |
| C3L\_02707 | C3L-02707 | C3L-02707 | C3L-02707 | C3L-02707 | Tumor | Classical |
| C3L\_02708 | C3L-02708 | C3L-02708 | C3L-02708 | C3L-02708 | Tumor | IDH mutant |
| C3L\_02900 | C3L-02900 | C3L-02900 | C3L-02900 | C3L-02900 | Tumor | IDH mutant |
| C3L\_02955 | C3L-02955 | C3L-02955 | NA | C3L-02955 | Tumor | Mesenchymal |
| C3L\_02970 | C3L-02970 | C3L-02970 | C3L-02970 | C3L-02970 | Tumor | Proneural |
| C3L\_02984 | C3L-02984 | C3L-02984 | C3L-02984 | C3L-02984 | Tumor | IDH mutant |
| C3L\_03260 | C3L-03260 | C3L-03260 | NA | C3L-03260 | Tumor | Proneural |
| C3L\_03266 | C3L-03266 | C3L-03266 | C3L-03266 | C3L-03266 | Tumor | Classical |
| C3L\_03387 | C3L-03387 | C3L-03387 | NA | C3L-03387 | Tumor | Classical |
| C3L\_03390 | C3L-03390 | C3L-03390 | NA | C3L-03390 | Tumor | Classical |
| C3L\_03392 | C3L-03392 | C3L-03392 | NA | C3L-03392 | Tumor | Mesenchymal |
| C3L\_03400 | C3L-03400 | C3L-03400 | NA | C3L-03400 | Tumor | Mesenchymal |
| C3L\_03405 | C3L-03405 | C3L-03405 | NA | C3L-03405 | Tumor | Proneural |
| C3L\_03407 | C3L-03407 | C3L-03407 | C3L-03407 | C3L-03407 | Tumor | Mesenchymal |
| C3L\_03681 | C3L-03681 | C3L-03681 | C3L-03681 | C3L-03681 | Tumor | Classical |
| C3L\_03727 | C3L-03727 | C3L-03727 | C3L-03727 | C3L-03727 | Tumor | Mesenchymal |
| C3L\_03728 | C3L-03728 | C3L-03728 | C3L-03728 | C3L-03728 | Tumor | Proneural |
| C3L\_03744 | C3L-03744 | C3L-03744 | NA | C3L-03744 | Tumor | Mesenchymal |
| C3L\_03748 | C3L-03748 | C3L-03748 | C3L-03748 | C3L-03748 | Tumor | Mesenchymal |
| C3L\_03968 | C3L-03968 | C3L-03968 | C3L-03968 | C3L-03968 | Tumor | Proneural |
| C3L\_04084 | C3L-04084 | C3L-04084 | C3L-04084 | C3L-04084 | Tumor | Classical |
| C3N\_00661 | C3N-00661 | C3N-00661 | C3N-00661 | C3N-00661 | Tumor | Mesenchymal |
| C3N\_00662 | C3N-00662 | C3N-00662 | C3N-00662 | C3N-00662 | Tumor | Proneural |
| C3N\_00663 | C3N-00663 | C3N-00663 | C3N-00663 | C3N-00663 | Tumor | IDH mutant |
| C3N\_00665 | C3N-00665 | C3N-00665 | C3N-00665 | C3N-00665 | Tumor | Mesenchymal |
| C3N\_01192 | C3N-01192 | C3N-01192 | C3N-01192 | C3N-01192 | Tumor | Mesenchymal |
| C3N\_01196 | C3N-01196 | C3N-01196 | C3N-01196 | C3N-01196 | Tumor | Classical |
| C3N\_01334 | C3N-01334 | C3N-01334 | C3N-01334 | C3N-01334 | Tumor | Classical |
| C3N\_01364 | C3N-01364 | C3N-01364 | C3N-01364 | C3N-01364 | Tumor | Mesenchymal |
| C3N\_01366 | C3N-01366 | C3N-01366 | C3N-01368 | C3N-01366 | Tumor | Mesenchymal |
| C3N\_01367 | C3N-01367 | C3N-01367 | NA | C3N-01367 | Tumor | Mesenchymal |
| C3N\_01368 | C3N-01368 | C3N-01368 | NA | C3N-01368 | Tumor | Classical |
| C3N\_01369 | C3N-01369 | C3N-01369 | C3N-01369 | C3N-01369 | Tumor | Mesenchymal |
| C3N\_01505 | C3N-01505 | C3N-01505 | C3N-01505 | C3N-01505 | Tumor | Mesenchymal |
| C3N\_01515 | C3N-01515 | C3N-01515 | C3N-01515 | C3N-01515 | Tumor | Mesenchymal |
| C3N\_01517 | C3N-01517 | C3N-01517 | C3N-01517 | C3N-01517 | Tumor | Classical |
| C3N\_01518 | C3N-01518 | C3N-01518 | C3N-01518 | C3N-01518 | Tumor | Mesenchymal |
| C3N\_01798 | C3N-01798 | C3N-01798 | C3N-01798 | C3N-01798 | Tumor | Proneural |
| C3N\_01814 | C3N-01814 | C3N-01814 | C3N-01814 | C3N-01814 | Tumor | Proneural |
| C3N\_01815 | C3N-01815 | C3N-01815 | C3N-01815 | C3N-01815 | Tumor | Mesenchymal |
| C3N\_01816 | C3N-01816 | C3N-01816 | C3N-01816 | C3N-01816 | Tumor | Mesenchymal |
| C3N\_01818 | C3N-01818 | C3N-01818 | C3N-01818 | C3N-01818 | Tumor | Proneural |
| C3N\_01851 | C3N-01851 | C3N-01851 | NA | C3N-01851 | Tumor | Proneural |
| C3N\_01852 | C3N-01852 | C3N-01852 | C3N-01852 | C3N-01852 | Tumor | Classical |
| C3N\_01856 | C3N-01856 | C3N-01856 | C3N-01856 | C3N-01856 | Tumor | Mesenchymal |
| C3N\_01857 | C3N-01857 | C3N-01857 | NA | C3N-01857 | Tumor | Classical |
| C3N\_02181 | C3N-02181 | C3N-02181 | C3N-02181 | C3N-02181 | Tumor | Mesenchymal |
| C3N\_02183 | C3N-02183 | C3N-02183 | C3N-02183 | C3N-02183 | Tumor | Mesenchymal |
| C3N\_02185 | C3N-02185 | C3N-02185 | C3N-02185 | C3N-02185 | Tumor | Proneural |
| C3N\_02186 | C3N-02186 | C3N-02186 | C3N-02186 | C3N-02186 | Tumor | Proneural |
| C3N\_02188 | C3N-02188 | C3N-02188 | C3N-02188 | C3N-02188 | Tumor | Mesenchymal |
| C3N\_02190 | C3N-02190 | C3N-02190 | C3N-02190 | C3N-02190 | Tumor | Mesenchymal |
| C3N\_02255 | C3N-02255 | C3N-02255 | C3N-02255 | C3N-02255 | Tumor | Proneural |
| C3N\_02256 | C3N-02256 | C3N-02256 | C3N-02256 | C3N-02256 | Tumor | Proneural |
| C3N\_02769 | C3N-02769 | C3N-02769 | C3N-02769 | C3N-02769 | Tumor | Mesenchymal |
| C3N\_02770 | C3N-02770 | C3N-02770 | C3N-02770 | C3N-02770 | Tumor | Proneural |
| C3N\_02782 | C3N-02782 | C3N-02782 | C3N-02782 | C3N-02782 | Tumor | Classical |
| C3N\_02783 | C3N-02783 | C3N-02783 | C3N-02783 | C3N-02783 | Tumor | Proneural |
| C3N\_02784 | C3N-02784 | C3N-02784 | C3N-02784 | C3N-02784 | Tumor | Mesenchymal |
| C3N\_02785 | C3N-02785 | C3N-02785 | C3N-02785 | C3N-02785 | Tumor | Proneural |
| C3N\_02786 | C3N-02786 | C3N-02786 | C3N-02786 | C3N-02786 | Tumor | Mesenchymal |
| C3N\_02788 | C3N-02788 | C3N-02788 | C3N-02788 | C3N-02788 | Tumor | Proneural |
| C3N\_03070 | C3N-03070 | C3N-03070 | C3N-03070 | C3N-03070 | Tumor | IDH mutant |
| C3N\_03088 | C3N-03088 | C3N-03088 | C3N-03088 | C3N-03088 | Tumor | Proneural |
| C3N\_03180 | C3N-03180 | C3N-03180 | C3N-03180 | C3N-03180 | Tumor | Mesenchymal |
| C3N\_03182 | C3N-03182 | C3N-03182 | C3N-03182 | C3N-03182 | Tumor | Mesenchymal |
| C3N\_03183 | C3N-03183 | C3N-03183 | C3N-03183 | C3N-03183 | Tumor | Mesenchymal |
| C3N\_03184 | C3N-03184 | C3N-03184 | C3N-03184 | C3N-03184 | Tumor | Classical |
| C3N\_03186 | C3N-03186 | C3N-03186 | C3N-03186 | C3N-03186 | Tumor | Proneural |
| C3N\_03188 | C3N-03188 | C3N-03188 | C3N-03188 | C3N-03188 | Tumor | Classical |
| C3N\_03473 | C3N-03473 | C3N-03473 | NA | C3N-03473 | Tumor | Proneural |
| PT\_NPJ7 | PT-NPJ7 | PT-NPJ7 | PT-NPJ7 | PT-NPJ7 | Healthy | Healthy |
| PT\_P44H | PT-P44H | PT-P44H | PT-P44H | PT-P44H | Healthy | Healthy |
| PT\_Q2AG | PT-Q2AG | PT-Q2AG | PT-Q2AG | NA | Healthy | Healthy |
| PT\_QVJO | PT-QVJO | PT-QVJO | NA | PT-QVJO | Healthy | Healthy |
| PT\_R55F | PT-R55F | PT-R55F | PT-R55F | PT-R55F | Healthy | Healthy |
| PT\_RN5K | PT-RN5K | PT-RN5K | PT-RN5K | PT-RN5K | Healthy | Healthy |
| PT\_RU72 | PT-RU72 | PT-RU72 | NA | PT-RU72 | Healthy | Healthy |
| PT\_UTHO | PT-UTHO | PT-UTHO | PT-UTHO | PT-UTHO | Healthy | Healthy |
| PT\_WVLH | PT-WVLH | PT-WVLH | PT-WVLH | PT-WVLH | Healthy | Healthy |
| PT\_Y8DK | PT-Y8DK | PT-Y8DK | NA | PT-Y8DK | Healthy | Healthy |

## Name

The name(s) of the datasets that were provided in the annotation file
under “Analysis Name”.

```
## [1] "prot"    "phospho" "rna"     "met"
```

## Format

The format of the data file(s) uploaded.

```
## [1] "ProteinGroups" "PhosphoSites"  "RNA"           "MetabolitePos"
```

## Contrasts

Contrasts chosen under the model design. Contrasts are formatted
“Group1-Group2” where any features up-regulated with regards to that
contrast are up-regulated in Group1 as compared to Group2.

```
## [1] "Tumor-Healthy"
```

## Model Equation

The format of the equation used to design the `limma`
linear model which is used for differential analysis.

```
## [1] "~ 0 + Group"
```

## Data Overview

An overview of the structure of the expression set(s) used for the
majority of analyses output.

```
## prot : 
##       Number of Samples: 109 
##       Sample Names: C3L_00104 C3L_00365 C3L_00674 C3L_00677 C3L_01040 C3L_01043 C3L_01045 C3L_01046 C3L_01048 C3L_01049 C3L_01061 C3L_01142 C3L_01146 C3L_01149 C3L_01154 C3L_01155 C3L_01156 C3L_01157 C3L_01327 C3L_01834 C3L_01887 C3L_02041 C3L_02465 C3L_02504 C3L_02542 C3L_02642 C3L_02704 C3L_02705 C3L_02707 C3L_02708 C3L_02900 C3L_02955 C3L_02970 C3L_02984 C3L_03260 C3L_03266 C3L_03387 C3L_03390 C3L_03392 C3L_03400 C3L_03405 C3L_03407 C3L_03681 C3L_03727 C3L_03728 C3L_03744 C3L_03748 C3L_03968 C3L_04084 C3N_00661 C3N_00662 C3N_00663 C3N_00665 C3N_01192 C3N_01196 C3N_01334 C3N_01364 C3N_01366 C3N_01367 C3N_01368 C3N_01369 C3N_01505 C3N_01515 C3N_01517 C3N_01518 C3N_01798 C3N_01814 C3N_01815 C3N_01816 C3N_01818 C3N_01851 C3N_01852 C3N_01856 C3N_01857 C3N_02181 C3N_02183 C3N_02185 C3N_02186 C3N_02188 C3N_02190 C3N_02255 C3N_02256 C3N_02769 C3N_02770 C3N_02782 C3N_02783 C3N_02784 C3N_02785 C3N_02786 C3N_02788 C3N_03070 C3N_03088 C3N_03180 C3N_03182 C3N_03183 C3N_03184 C3N_03186 C3N_03188 C3N_03473 PT_NPJ7 PT_P44H PT_Q2AG PT_QVJO PT_R55F PT_RN5K PT_RU72 PT_UTHO PT_WVLH PT_Y8DK 
##       Number of Features in Raw Data: 10057 
##       Number of Features in Processed Data: 10057 
##       Number of Missing Values in Raw Data: 21751 
##       Number of Missing Features in Processed Data: 21751 
## 
## 
## phospho : 
##       Number of Samples: 109 
##       Sample Names: C3L_00104 C3L_00365 C3L_00674 C3L_00677 C3L_01040 C3L_01043 C3L_01045 C3L_01046 C3L_01048 C3L_01049 C3L_01061 C3L_01142 C3L_01146 C3L_01149 C3L_01154 C3L_01155 C3L_01156 C3L_01157 C3L_01327 C3L_01834 C3L_01887 C3L_02041 C3L_02465 C3L_02504 C3L_02542 C3L_02642 C3L_02704 C3L_02705 C3L_02707 C3L_02708 C3L_02900 C3L_02955 C3L_02970 C3L_02984 C3L_03260 C3L_03266 C3L_03387 C3L_03390 C3L_03392 C3L_03400 C3L_03405 C3L_03407 C3L_03681 C3L_03727 C3L_03728 C3L_03744 C3L_03748 C3L_03968 C3L_04084 C3N_00661 C3N_00662 C3N_00663 C3N_00665 C3N_01192 C3N_01196 C3N_01334 C3N_01364 C3N_01366 C3N_01367 C3N_01368 C3N_01369 C3N_01505 C3N_01515 C3N_01517 C3N_01518 C3N_01798 C3N_01814 C3N_01815 C3N_01816 C3N_01818 C3N_01851 C3N_01852 C3N_01856 C3N_01857 C3N_02181 C3N_02183 C3N_02185 C3N_02186 C3N_02188 C3N_02190 C3N_02255 C3N_02256 C3N_02769 C3N_02770 C3N_02782 C3N_02783 C3N_02784 C3N_02785 C3N_02786 C3N_02788 C3N_03070 C3N_03088 C3N_03180 C3N_03182 C3N_03183 C3N_03184 C3N_03186 C3N_03188 C3N_03473 PT_NPJ7 PT_P44H PT_Q2AG PT_QVJO PT_R55F PT_RN5K PT_RU72 PT_UTHO PT_WVLH PT_Y8DK 
##       Number of Features in Raw Data: 17227 
##       Number of Features in Processed Data: 17227 
##       Number of Missing Values in Raw Data: 216680 
##       Number of Missing Features in Processed Data: 216680 
## 
## 
## rna : 
##       Number of Samples: 108 
##       Sample Names: C3L_00104 C3L_00365 C3L_00674 C3L_00677 C3L_01040 C3L_01043 C3L_01045 C3L_01046 C3L_01048 C3L_01049 C3L_01061 C3L_01142 C3L_01146 C3L_01149 C3L_01154 C3L_01155 C3L_01156 C3L_01157 C3L_01327 C3L_01834 C3L_01887 C3L_02041 C3L_02465 C3L_02504 C3L_02542 C3L_02642 C3L_02704 C3L_02705 C3L_02707 C3L_02708 C3L_02900 C3L_02955 C3L_02970 C3L_02984 C3L_03260 C3L_03266 C3L_03387 C3L_03390 C3L_03392 C3L_03400 C3L_03405 C3L_03407 C3L_03681 C3L_03727 C3L_03728 C3L_03744 C3L_03748 C3L_03968 C3L_04084 C3N_00661 C3N_00662 C3N_00663 C3N_00665 C3N_01192 C3N_01196 C3N_01334 C3N_01364 C3N_01366 C3N_01367 C3N_01368 C3N_01369 C3N_01505 C3N_01515 C3N_01517 C3N_01518 C3N_01798 C3N_01814 C3N_01815 C3N_01816 C3N_01818 C3N_01851 C3N_01852 C3N_01856 C3N_01857 C3N_02181 C3N_02183 C3N_02185 C3N_02186 C3N_02188 C3N_02190 C3N_02255 C3N_02256 C3N_02769 C3N_02770 C3N_02782 C3N_02783 C3N_02784 C3N_02785 C3N_02786 C3N_02788 C3N_03070 C3N_03088 C3N_03180 C3N_03182 C3N_03183 C3N_03184 C3N_03186 C3N_03188 C3N_03473 PT_NPJ7 PT_P44H PT_QVJO PT_R55F PT_RN5K PT_RU72 PT_UTHO PT_WVLH PT_Y8DK 
##       Number of Features in Raw Data: 17018 
##       Number of Features in Processed Data: 17018 
##       Number of Missing Values in Raw Data: 21452 
##       Number of Missing Features in Processed Data: 21452 
## 
## 
## met : 
##       Number of Samples: 82 
##       Sample Names: C3L_00365 C3L_00674 C3L_00677 C3L_01043 C3L_01045 C3L_01048 C3L_01049 C3L_01149 C3L_01157 C3L_01887 C3L_02041 C3L_02465 C3L_02504 C3L_02542 C3L_02642 C3L_02704 C3L_02705 C3L_02707 C3L_02708 C3L_02900 C3L_02970 C3L_02984 C3L_03266 C3L_03407 C3L_03681 C3L_03727 C3L_03728 C3L_03748 C3L_03968 C3L_04084 C3N_00661 C3N_00662 C3N_00663 C3N_00665 C3N_01192 C3N_01196 C3N_01334 C3N_01364 C3N_01366 C3N_01369 C3N_01505 C3N_01515 C3N_01517 C3N_01518 C3N_01798 C3N_01814 C3N_01815 C3N_01816 C3N_01818 C3N_01852 C3N_01856 C3N_02181 C3N_02183 C3N_02185 C3N_02186 C3N_02188 C3N_02190 C3N_02255 C3N_02256 C3N_02769 C3N_02770 C3N_02782 C3N_02783 C3N_02784 C3N_02785 C3N_02786 C3N_02788 C3N_03070 C3N_03088 C3N_03180 C3N_03182 C3N_03183 C3N_03184 C3N_03186 C3N_03188 PT_NPJ7 PT_P44H PT_Q2AG PT_R55F PT_RN5K PT_UTHO PT_WVLH 
##       Number of Features in Raw Data: 119 
##       Number of Features in Processed Data: 119 
##       Number of Missing Values in Raw Data: 62 
##       Number of Missing Features in Processed Data: 62
```

The below statistical overview(s) provide information on mean, range,
and variance measures.

### Stats: prot

### Stats: phospho

### Stats: rna

### Stats: met

## Missingness

The below plot(s) are generated using the VIM package to look at the
spread of missing values across the samples in each dataset. This is
generated using pre-normalized/filtered data. The right half of the plot
shows the different combinations of groups that contain missing values
in the same features, with the bottom row showing how many features are
not missing in *any* sample.

```
## prot missing value overview:
```

```
## phospho missing value overview:
```

```
## rna missing value overview:
```

```
## met missing value overview:
```

# Normalization

Normalization plots show how the data changes pre and post
normalization. If no normalization method was chosen, only one plot will
be generated. Additional normalization visualizations - MA plots,
Relative Log Expression plots, and QQ plots - can be found in the
associated image download folder.

# PCA Plot

Principal Component Analysis is a useful tool for assessing
similarity among factors of a high dimensional data set.

# UMAP

UMAP, or Uniform Manifold Approximation and Projection, is used for
dimensionality reduction and data visualization. It is commonly compared
to its predecessor, t-SNE or t-distributed Stochastic Neighbor
Embedding, but is generally much faster.

# MD Plots

Mean-Difference plots are used to visualize log-intensity ratios, or
‘differences’, versus log-intensity averages, or ‘means’.

# Correlation Plots

The correlation plots show correlation values among the groups of the
data set. The legend on the right shows the color scale over the range
of correlation values. Please note that the correlation plots are
assessing similarity over overall sample intensities.

# Heatmap

This heatmap shows an overview of the relative feature intensity
across the dataset(s) before linear modeling.

# Volcano Plots

The volcano plots are generated based on the options selected for the
linear model. Each contrast (or if contrasts are not selected each
group) has its own correlating volcano plot. The volcano plots show log
fold change values on the x-axis and log p-values or adjusted p-values
on the y-axis.

### Adjusted P-Value

### P-Value

# Differential Heatmaps

The differential heatmaps are based on the output of the limma linear
model. Each contrast or group has a corresponding heatmap, which is
subset by the top 50 most variable rows/genes.

# Enrichment

## Parameters

### Species

The selected species that correlated with the data input.

```
## human
```

### Enrichment Type

Either ranked or unranked enrichment analysis.

```
## Ranked Enrichment Analysis
```

```
## Gene Lists:
```

```
## List of 3
##  $ prot_GSEA    :List of 1
##   ..$ Tumor-Healthy: Named num [1:10057] 30.5 30 28.4 28.1 27.7 ...
##   .. ..- attr(*, "names")= chr [1:10057] "VIM" "ACTL6A" "ALB" "SEC11A" ...
##  $ phospho_GSEA :List of 1
##   ..$ Tumor-Healthy: Named num [1:4965] 41.1 32.3 31.9 30.3 29.8 ...
##   .. ..- attr(*, "names")= chr [1:4965] "GBF1" "SPAG9" "CANX" "HTT" ...
##  $ phospho_PKSEA:List of 1
##   ..$ Tumor-Healthy: Named num [1:16913] 41.1 32.3 31.9 30.3 29.8 ...
##   .. ..- attr(*, "names")= chr [1:16913] "Q92538;S233" "O60271;T217" "NA;S637" "P42858;S642" ...
## NULL
```

```
## Enrichmed Pathways Present?
```

```
## prot_Tumor-Healthy HUMAN_KEGG : TRUE 
## prot_Tumor-Healthy HUMAN_Reactome : TRUE 
## prot_Tumor-Healthy HUMAN_WikiPathways : TRUE 
## prot_Tumor-Healthy HUMAN_GOBP : TRUE 
## prot_Tumor-Healthy HUMAN_GOCC : TRUE 
## prot_Tumor-Healthy HUMAN_GOMF : TRUE 
## prot_Tumor-Healthy HUMAN_Hallmark : TRUE 
## prot_Tumor-Healthy HUMAN_MSigDB : TRUE 
## prot_Tumor-Healthy HUMAN_MOMENTABiocycEXP : TRUE 
## prot_Tumor-Healthy HUMAN_MOMENTAMFNEXP : TRUE 
## phospho_Tumor-Healthy HUMAN_KEGG : TRUE 
## phospho_Tumor-Healthy HUMAN_Reactome : TRUE 
## phospho_Tumor-Healthy HUMAN_WikiPathways : TRUE 
## phospho_Tumor-Healthy HUMAN_GOBP : TRUE 
## phospho_Tumor-Healthy HUMAN_GOCC : TRUE 
## phospho_Tumor-Healthy HUMAN_GOMF : TRUE 
## phospho_Tumor-Healthy HUMAN_Hallmark : TRUE 
## phospho_Tumor-Healthy HUMAN_MSigDB : TRUE 
## phospho_Tumor-Healthy HUMAN_MOMENTABiocycEXP : TRUE 
## phospho_Tumor-Healthy HUMAN_MOMENTAMFNEXP : TRUE 
## phospho_Tumor-Healthy HUMAN_PHOSPHO_UNIPROT : TRUE
```

## Dot Plot

The dot plot depicts the enrichment scores (p-values) via color-coded
points, and gene ratio as position relative to the x-axis. Gene count is
represented by the size of the point.

## EMAP Plot

According to the Yu lab, “the enrichment map organizes enriched terms
into a network with edges connecting overlapping gene sets. In this way,
mutually overlapping gene sets are tend to cluster together, making it
easy to identify functional modules.”

# S-Score Integration

### S-Score Integration: prot\_phospho\_rna\_met\_Tumor-Healthy

## S-Score Enrichment

### Dot Plot

The dot plot depicts the enrichment scores (p-values) via color-coded
points, and gene ratio as position relative to the x-axis. Gene count is
represented by the size of the point.

### EMAP Plot

According to the Yu lab, “the enrichment map organizes enriched terms
into a network with edges connecting overlapping gene sets. In this way,
mutually overlapping gene sets are tend to cluster together, making it
easy to identify functional modules.”

# PCSF

```
## PCSF Datasets/Contrasts Generated: prot_Tumor-Healthy; phospho_Tumor-Healthy; rna_Tumor-Healthy; Sscore_prot_phospho_rna_met_Tumor-Healthy
```

### Node Network: prot\_Tumor-Healthy

visNetwork

### Node Network: phospho\_Tumor-Healthy

visNetwork

### Node Network: rna\_Tumor-Healthy

visNetwork

### Node Network: Sscore\_prot\_phospho\_rna\_met\_Tumor-Healthy

visNetwork

### Influential Network: prot\_Tumor-Healthy

visNetwork

### Influential Network: phospho\_Tumor-Healthy

visNetwork

### Influential Network: rna\_Tumor-Healthy

visNetwork

### Influential Network: Sscore\_prot\_phospho\_rna\_met\_Tumor-Healthy

visNetwork

## PCSF Enrichment

### PCSF Enriched: prot\_Tumor-Healthy

```
## Error in name2int(valueCols, ...): You specified the columns: Adj_PVal, but the column names of the data are ID, Description, GeneRatio, BgRatio, RichFactor, FoldEnrichment, zScore, pvalue, p.adjust, qvalue, geneID, Count
```

### Enriched Network: prot\_Tumor-Healthy

visNetwork

### Enriched Network: phospho\_Tumor-Healthy

visNetwork

### Enriched Network: rna\_Tumor-Healthy

visNetwork

### Enriched Network: Sscore\_prot\_phospho\_rna\_met\_Tumor-Healthy

visNetwork

# Parameters

```
## File Name(s):  prot; phospho; rna; met 
##  File Format(s):  ProteinGroups; PhosphoSites; RNA; MetabolitePos 
##  Species:  human 
##  Data Cleaning, Filtering, & Transformation: 
##       Normalization:  none 
##       Log Transformation:  FALSE 
##       Percent Zeros Filter:  30 % 
##       Missing Value Imputation:  FALSE 
##       Uniprot Annotation:  TRUE 
##       Group By:  Group 
##  Data Modeling: 
##       Contrasts:  Tumor-Healthy 
##       Linear Model:  ~ 0 + Group 
##  Enrichment Performed on Linear Model:  TRUE 
##       Enrichment Algorithm:  ranked
```

# Session Info

See information about the system that generated this report
below.

```
## R version 4.4.1 (2024-06-14)
## Platform: x86_64-apple-darwin20
## Running under: macOS Sonoma 14.6.1
## 
## Matrix products: default
## BLAS:   /System/Library/Frameworks/Accelerate.framework/Versions/A/Frameworks/vecLib.framework/Versions/A/libBLAS.dylib 
## LAPACK: /Library/Frameworks/R.framework/Versions/4.4-x86_64/Resources/lib/libRlapack.dylib;  LAPACK version 3.12.0
## 
## locale:
## [1] en_US.UTF-8/en_US.UTF-8/en_US.UTF-8/C/en_US.UTF-8/en_US.UTF-8
## 
## time zone: America/Los_Angeles
## tzcode source: internal
## 
## attached base packages:
## [1] stats     graphics  grDevices utils     datasets  methods   base     
## 
## other attached packages:
##  [1] kableExtra_1.4.0    pathview_1.44.0     NormalyzerDE_1.22.0 Biobase_2.64.0      BiocGenerics_0.50.0 lubridate_1.9.3     forcats_1.0.0      
##  [8] stringr_1.5.1       dplyr_1.1.4         purrr_1.0.2         readr_2.1.5         tidyr_1.3.1         tibble_3.2.1        ggplot2_3.5.1      
## [15] tidyverse_2.0.0     shiny_1.9.1        
## 
## loaded via a namespace (and not attached):
##   [1] IRanges_2.38.1              R.methodsS3_1.8.2           vroom_1.6.5                 pacman_0.5.1                nnet_7.3-19                
##   [6] DT_0.33                     Biostrings_2.72.1           vctrs_0.6.5                 digest_0.6.37               png_0.1-8                  
##  [11] shape_1.4.6.1               proxy_0.4-27                ggrepel_0.9.6               colourvalues_0.3.9          magick_2.8.4               
##  [16] MASS_7.3-60.2               reshape2_1.4.4              httpuv_1.6.15               foreach_1.5.2               qvalue_2.36.0              
##  [21] withr_3.0.2                 psych_2.4.6.26              xfun_0.48                   ggfun_0.1.6                 ggpubr_0.6.0               
##  [26] EnsDb.Hsapiens.v86_2.99.0   memoise_2.0.1               commonmark_1.9.1            clusterProfiler_4.12.6      gson_0.1.0                 
##  [31] systemfonts_1.1.0           ragg_1.3.2                  tidytree_0.4.6              zoo_1.8-12                  GlobalOptions_0.1.2        
##  [36] KEGGgraph_1.64.0            R.oo_1.26.0                 DEoptimR_1.1-3              KEGGREST_1.44.1             promises_1.3.0             
##  [41] httr_1.4.7                  rstatix_0.7.2               restfulr_0.0.15             rstudioapi_0.16.0           shinyAce_0.4.2             
##  [46] UCSC.utils_1.0.0            miniUI_0.1.1.1              generics_0.1.3              DOSE_3.30.5                 curl_6.0.0                 
##  [51] S4Vectors_0.42.1            zlibbioc_1.50.0             ggraph_2.2.1                polyclip_1.10-7             GenomeInfoDbData_1.2.12    
##  [56] SparseArray_1.4.8           xtable_1.8-4                doParallel_1.0.17           evaluate_0.24.0             S4Arrays_1.4.1             
##  [61] preprocessCore_1.66.0       hms_1.1.3                   GenomicRanges_1.56.1        irlba_2.3.5.1               colorspace_2.1-1           
##  [66] visNetwork_2.1.2            shinyWidgets_0.8.6          magrittr_2.0.3              lmtest_0.9-40               Rgraphviz_2.48.0           
##  [71] later_1.3.2                 viridis_0.6.5               ggtree_3.12.0               lattice_0.22-6              robustbase_0.99-4          
##  [76] SparseM_1.84-2              XML_3.99-0.17               shadowtext_0.1.4            cowplot_1.1.3               matrixStats_1.4.1          
##  [81] class_7.3-23                pillar_1.9.0                nlme_3.1-164                iterators_1.0.14            compiler_4.4.1             
##  [86] stringi_1.8.4               shinycssloaders_1.1.0       SummarizedExperiment_1.34.0 dendextend_1.17.1           GenomicAlignments_1.40.0   
##  [91] plyr_1.8.9                  crayon_1.5.3                abind_1.4-5                 BiocIO_1.14.0               gridGraphics_0.5-1         
##  [96] locfit_1.5-9.10             GeneTonic_2.8.0             sp_2.1-4                    graphlayouts_1.1.1          org.Hs.eg.db_3.19.1        
## [101] bit_4.0.5                   fastmatch_1.1-4             codetools_0.2-20            textshaping_0.4.0           crosstalk_1.2.1            
## [106] bslib_0.8.0                 e1071_1.7-14                GetoptLong_1.0.5            plotly_4.10.4               mime_0.12                  
## [111] splines_4.4.1               markdown_1.13               circlize_0.4.16             Rcpp_1.0.13                 duckdb_1.3.3               
## [116] tippy_0.1.0                 knitr_1.48                  blob_1.2.4                  utf8_1.2.4                  clue_0.3-65                
## [121] AnnotationFilter_1.28.0     fs_1.6.4                    backbone_2.1.4              expm_1.0-0                  openxlsx_4.2.7             
## [126] ggsignif_0.6.4              ggplotify_0.1.2             Matrix_1.7-0                statmod_1.5.0               tzdb_0.4.0                 
## [131] svglite_2.1.3               tweenr_2.0.3                pkgconfig_2.0.3             pheatmap_1.0.13             tools_4.4.1                
## [136] cachem_1.1.0                RSQLite_2.3.7               viridisLite_0.4.2           DBI_1.2.3                   bsicons_0.1.2              
## [141] fastmap_1.2.0               rmarkdown_2.28              scales_1.3.0                grid_4.4.1                  Rsamtools_2.20.0           
## [146] broom_1.0.6                 sass_0.4.9                  patchwork_1.2.0             FNN_1.1.4                   graph_1.82.0               
## [151] carData_3.0-5               fontawesome_0.5.2           farver_2.1.2                tidygraph_1.3.1             scatterpie_0.2.4           
## [156] yaml_2.3.10                 MatrixGenerics_1.16.0       influential_2.2.9           rtracklayer_1.64.0          cli_3.6.3                  
## [161] stats4_4.4.1                lifecycle_1.0.4             rsconnect_1.3.1             uwot_0.2.2                  backports_1.5.0            
## [166] rintrojs_0.3.4              BiocParallel_1.38.0         timechange_0.3.0            gtable_0.3.6                rjson_0.2.23               
## [171] ggridges_0.5.6              parallel_4.4.1              ape_5.8                     limma_3.60.4                jsonlite_1.8.9             
## [176] colourpicker_1.3.0          bitops_1.0-8                bit64_4.0.5                 yulab.utils_0.1.7           zip_2.3.1                  
## [181] ranger_0.16.0               bs4Dash_2.3.4               jquerylib_0.1.4             highr_0.11                  GOSemSim_2.30.2            
## [186] shinyjs_2.1.0               R.utils_2.12.3              lazyeval_0.2.2              dynamicTreeCut_1.63-1       htmltools_0.5.8.1          
## [191] enrichplot_1.24.4           GO.db_3.19.1                rappdirs_0.3.3              ensembldb_2.28.1            glue_1.8.0                 
## [196] ggvenn_0.1.10               httr2_1.0.5                 XVector_0.44.0              VIM_6.2.2                   RCurl_1.98-1.16            
## [201] treeio_1.28.0               ComplexUpset_1.3.3          BH_1.84.0-0                 mnormt_2.1.1                gridExtra_2.3              
## [206] boot_1.3-30                 igraph_2.0.3                R6_2.5.1                    DESeq2_1.44.0               vcd_1.4-12                 
## [211] labeling_0.4.3              GenomicFeatures_1.56.0      cluster_2.1.6               aplot_0.2.3                 GenomeInfoDb_1.40.1        
## [216] DelayedArray_0.30.1         tidyselect_1.2.1            ProtGenerics_1.36.0         ggforce_0.4.2               xml2_1.3.6                 
## [221] car_3.1-2                   AnnotationDbi_1.66.0        munsell_0.5.1               laeken_0.5.3                topGO_2.56.0               
## [226] data.table_1.16.0           htmlwidgets_1.6.4           fgsea_1.30.0                ComplexHeatmap_2.20.0       RColorBrewer_1.1-3         
## [231] PCSF_0.99.1                 rlang_1.1.4                 ggnewscale_0.5.1            fansi_1.0.6                 Cairo_1.6-2
```
