# Supplementary material for: OmNI: a modular open-source framework for interactive multi-omics data integration and visualization
Source: NAR Genom Bioinform. 2026 Jan 10;8(1):lqaf206. doi: 10.1093/nargab/lqaf206 (PMC12789801; doi:10.1093/nargab/lqaf206)
Supplement: lqaf206_Supplemental_Files [file lqaf206_supplemental_files.zip › Fentanyl_OmNI_Report.html.zip!/Fentanyl_OmNI_Report.html]

OmNI Report


# OmNI Report

#### May 07, 2025

## Image Folder

```
## [1] "~/Downloads/Ratio_log_loess_OmNI_Report_2505070937"
```

> Right-click on any images to open them in a new tab, save them to
> your computer, or copy them.

# Dataset Information

An overview of the dataset uploaded and groups, contrasts, and
expression set used in generating the analyses in the report.

## Annotation File

| SampleName | Ratio\_Prot | Ratio\_Phos\_MS | Abund\_Prot | Abund\_Phos\_MS | Group | Drug | Plex | Strain | Session |
| --- | --- | --- | --- | --- | --- | --- | --- | --- | --- |
| F\_Svlmj\_1 | Fentanyl\_Svlmj\_P1 | Fentanyl\_Svlmj\_P1 | Fentanyl\_Svlmj\_P1 | Fentanyl\_Svlmj\_P1 | Fentanyl | Fentanyl | Plex1 | Svlmj | Session1 |
| F\_Aj\_1 | Fentanyl\_Aj\_P1 | Fentanyl\_Aj\_P1 | Fentanyl\_Aj\_P1 | Fentanyl\_Aj\_P1 | Fentanyl | Fentanyl | Plex1 | Aj | Session1 |
| F\_6j\_1 | Fentanyl\_6j\_P1 | Fentanyl\_6j\_P1 | Fentanyl\_6j\_P1 | Fentanyl\_6j\_P1 | Fentanyl | Fentanyl | Plex1 | 6j | Session1 |
| F\_CastEij\_1 | Fentanyl\_CastEij\_P1 | Fentanyl\_CastEij\_P1 | Fentanyl\_CastEij\_P1 | Fentanyl\_CastEij\_P1 | Fentanyl | Fentanyl | Plex1 | CastEij | Session4 |
| F\_2j\_1 | Fentanyl\_2j\_P1 | Fentanyl\_2j\_P1 | Fentanyl\_2j\_P1 | Fentanyl\_2j\_P1 | Fentanyl | Fentanyl | Plex1 | 2j | Session1 |
| F\_Shiltj\_1 | Fentanyl\_Shiltj\_P1 | Fentanyl\_Shiltj\_P1 | Fentanyl\_Shiltj\_P1 | Fentanyl\_Shiltj\_P1 | Fentanyl | Fentanyl | Plex1 | Shiltj | Session3 |
| F\_Hiltj\_1 | Fentanyl\_Hiltj\_P1 | Fentanyl\_Hiltj\_P1 | Fentanyl\_Hiltj\_P1 | Fentanyl\_Hiltj\_P1 | Fentanyl | Fentanyl | Plex1 | Hiltj | Session1 |
| F\_Phj\_1 | Fentanyl\_Phj\_P1 | Fentanyl\_Phj\_P1 | Fentanyl\_Phj\_P1 | Fentanyl\_Phj\_P1 | Fentanyl | Fentanyl | Plex1 | Phj | Session1 |
| F\_WsbEij\_1 | Fentanyl\_WsbEij\_P1 | Fentanyl\_WsbEij\_P1 | Fentanyl\_WsbEij\_P1 | Fentanyl\_WsbEij\_P1 | Fentanyl | Fentanyl | Plex1 | WsbEij | Session1 |
| S\_Svlmj\_1 | Saline\_Svlmj\_P1 | Saline\_Svlmj\_P1 | Saline\_Svlmj\_P1 | Saline\_Svlmj\_P1 | Saline | Saline | Plex1 | Svlmj | Session1 |
| S\_Aj\_1 | Saline\_Aj\_P1 | Saline\_Aj\_P1 | Saline\_Aj\_P1 | Saline\_Aj\_P1 | Saline | Saline | Plex1 | Aj | Session4 |
| S\_6j\_1 | Saline\_6j\_P1 | Saline\_6j\_P1 | Saline\_6j\_P1 | Saline\_6j\_P1 | Saline | Saline | Plex1 | 6j | Session1 |
| S\_CastEij\_1 | Saline\_CastEij\_P1 | Saline\_CastEij\_P1 | Saline\_CastEij\_P1 | Saline\_CastEij\_P1 | Saline | Saline | Plex1 | CastEij | Session2 |
| S\_2j\_1 | Saline\_2j\_P1 | Saline\_2j\_P1 | Saline\_2j\_P1 | Saline\_2j\_P1 | Saline | Saline | Plex1 | 2j | Session4 |
| S\_Shiltj\_1 | Saline\_Shiltj\_P1 | Saline\_Shiltj\_P1 | Saline\_Shiltj\_P1 | Saline\_Shiltj\_P1 | Saline | Saline | Plex1 | Shiltj | Session4 |
| S\_Hiltj\_1 | Saline\_Hiltj\_P1 | Saline\_Hiltj\_P1 | Saline\_Hiltj\_P1 | Saline\_Hiltj\_P1 | Saline | Saline | Plex1 | Hiltj | Session1 |
| S\_Phj\_1 | Saline\_Phj\_P1 | Saline\_Phj\_P1 | Saline\_Phj\_P1 | Saline\_Phj\_P1 | Saline | Saline | Plex1 | Phj | Session3 |
| S\_WsbEij\_1 | Saline\_WsbEij\_P1 | Saline\_WsbEij\_P1 | Saline\_WsbEij\_P1 | Saline\_WsbEij\_P1 | Saline | Saline | Plex1 | WsbEij | Session3 |
| F\_Svlmj\_2 | Fentanyl\_Svlmj\_P2 | Fentanyl\_Svlmj\_P2 | Fentanyl\_Svlmj\_P2 | Fentanyl\_Svlmj\_P2 | Fentanyl | Fentanyl | Plex2 | Svlmj | Session3 |
| F\_Aj\_2 | Fentanyl\_Aj\_P2 | Fentanyl\_Aj\_P2 | Fentanyl\_Aj\_P2 | Fentanyl\_Aj\_P2 | Fentanyl | Fentanyl | Plex2 | Aj | Session4 |
| F\_6j\_2 | Fentanyl\_6j\_P2 | Fentanyl\_6j\_P2 | Fentanyl\_6j\_P2 | Fentanyl\_6j\_P2 | Fentanyl | Fentanyl | Plex2 | 6j | Session2 |
| F\_CastEij\_2 | Fentanyl\_CastEij\_P2 | Fentanyl\_CastEij\_P2 | Fentanyl\_CastEij\_P2 | Fentanyl\_CastEij\_P2 | Fentanyl | Fentanyl | Plex2 | CastEij | Session2 |
| F\_2j\_2 | Fentanyl\_2j\_P2 | Fentanyl\_2j\_P2 | Fentanyl\_2j\_P2 | Fentanyl\_2j\_P2 | Fentanyl | Fentanyl | Plex2 | 2j | Session3 |
| F\_Shiltj\_2 | Fentanyl\_Shiltj\_P2 | Fentanyl\_Shiltj\_P2 | Fentanyl\_Shiltj\_P2 | Fentanyl\_Shiltj\_P2 | Fentanyl | Fentanyl | Plex2 | Shiltj | Session2 |
| F\_Hiltj\_2 | Fentanyl\_Hiltj\_P2 | Fentanyl\_Hiltj\_P2 | Fentanyl\_Hiltj\_P2 | Fentanyl\_Hiltj\_P2 | Fentanyl | Fentanyl | Plex2 | Hiltj | Session3 |
| F\_Phj\_2 | Fentanyl\_Phj\_P2 | Fentanyl\_Phj\_P2 | Fentanyl\_Phj\_P2 | Fentanyl\_Phj\_P2 | Fentanyl | Fentanyl | Plex2 | Phj | Session2 |
| F\_WsbEij\_2 | Fentanyl\_WsbEij\_P2 | Fentanyl\_WsbEij\_P2 | Fentanyl\_WsbEij\_P2 | Fentanyl\_WsbEij\_P2 | Fentanyl | Fentanyl | Plex2 | WsbEij | Session2 |
| S\_Svlmj\_2 | Saline\_Svlmj\_P2 | Saline\_Svlmj\_P2 | Saline\_Svlmj\_P2 | Saline\_Svlmj\_P2 | Saline | Saline | Plex2 | Svlmj | Session4 |
| S\_Aj\_2 | Saline\_Aj\_P2 | Saline\_Aj\_P2 | Saline\_Aj\_P2 | Saline\_Aj\_P2 | Saline | Saline | Plex2 | Aj | Session3 |
| S\_6j\_2 | Saline\_6j\_P2 | Saline\_6j\_P2 | Saline\_6j\_P2 | Saline\_6j\_P2 | Saline | Saline | Plex2 | 6j | Session3 |
| S\_CastEij\_2 | Saline\_CastEij\_P2 | Saline\_CastEij\_P2 | Saline\_CastEij\_P2 | Saline\_CastEij\_P2 | Saline | Saline | Plex2 | CastEij | Session3 |
| S\_2j\_2 | Saline\_2j\_P2 | Saline\_2j\_P2 | Saline\_2j\_P2 | Saline\_2j\_P2 | Saline | Saline | Plex2 | 2j | Session2 |
| S\_Shiltj\_2 | Saline\_Shiltj\_P2 | Saline\_Shiltj\_P2 | Saline\_Shiltj\_P2 | Saline\_Shiltj\_P2 | Saline | Saline | Plex2 | Shiltj | Session2 |
| S\_Hiltj\_2 | Saline\_Hiltj\_P2 | Saline\_Hiltj\_P2 | Saline\_Hiltj\_P2 | Saline\_Hiltj\_P2 | Saline | Saline | Plex2 | Hiltj | Session3 |
| S\_Phj\_2 | Saline\_Phj\_P2 | Saline\_Phj\_P2 | Saline\_Phj\_P2 | Saline\_Phj\_P2 | Saline | Saline | Plex2 | Phj | Session2 |
| S\_WsbEij\_2 | Saline\_WsbEij\_P2 | Saline\_WsbEij\_P2 | Saline\_WsbEij\_P2 | Saline\_WsbEij\_P2 | Saline | Saline | Plex2 | WsbEij | Session4 |

## Name

The name(s) of the datasets that were provided in the annotation file
under “Analysis Name”.

```
## [1] "Ratio_Prot"    "Ratio_Phos_MS"
```

## Format

The format of the data file(s) uploaded.

```
## [1] "ProteinGroups" "PhosphoSites"
```

## Contrasts

Contrasts chosen under the model design. Contrasts are formatted
“Group1-Group2” where any features up-regulated with regards to that
contrast are up-regulated in Group1 as compared to Group2.

```
## [1] "Fentanyl-Saline"
```

## Model Equation

The format of the equation used to design the `limma`
linear model which is used for differential analysis.

```
## [1] "~ 0 + Drug + Session"
```

## Data Overview

An overview of the structure of the expression set(s) used for the
majority of analyses output.

```
## Ratio_Prot : 
##       Number of Samples: 36 
##       Sample Names: F_Svlmj_1 F_Aj_1 F_6j_1 F_CastEij_1 F_2j_1 F_Shiltj_1 F_Hiltj_1 F_Phj_1 F_WsbEij_1 S_Svlmj_1 S_Aj_1 S_6j_1 S_CastEij_1 S_2j_1 S_Shiltj_1 S_Hiltj_1 S_Phj_1 S_WsbEij_1 F_Svlmj_2 F_Aj_2 F_6j_2 F_CastEij_2 F_2j_2 F_Shiltj_2 F_Hiltj_2 F_Phj_2 F_WsbEij_2 S_Svlmj_2 S_Aj_2 S_6j_2 S_CastEij_2 S_2j_2 S_Shiltj_2 S_Hiltj_2 S_Phj_2 S_WsbEij_2 
##       Number of Features in Raw Data: 9287 
##       Number of Features in Processed Data: 9287 
##       Number of Missing Values in Raw Data: 152 
##       Number of Missing Features in Processed Data: 0 
## 
## 
## Ratio_Phos_MS : 
##       Number of Samples: 36 
##       Sample Names: F_Svlmj_1 F_Aj_1 F_6j_1 F_CastEij_1 F_2j_1 F_Shiltj_1 F_Hiltj_1 F_Phj_1 F_WsbEij_1 S_Svlmj_1 S_Aj_1 S_6j_1 S_CastEij_1 S_2j_1 S_Shiltj_1 S_Hiltj_1 S_Phj_1 S_WsbEij_1 F_Svlmj_2 F_Aj_2 F_6j_2 F_CastEij_2 F_2j_2 F_Shiltj_2 F_Hiltj_2 F_Phj_2 F_WsbEij_2 S_Svlmj_2 S_Aj_2 S_6j_2 S_CastEij_2 S_2j_2 S_Shiltj_2 S_Hiltj_2 S_Phj_2 S_WsbEij_2 
##       Number of Features in Raw Data: 12333 
##       Number of Features in Processed Data: 12333 
##       Number of Missing Values in Raw Data: 21 
##       Number of Missing Features in Processed Data: 0
```

The below statistical overview(s) provide information on mean, range,
and variance measures.

### Stats: Ratio\_Prot

### Stats: Ratio\_Phos\_MS

## Missingness

The below plot(s) are generated using the VIM package to look at the
spread of missing values across the samples in each dataset. This is
generated using pre-normalized/filtered data. The right half of the plot
shows the different combinations of groups that contain missing values
in the same features, with the bottom row showing how many features are
not missing in *any* sample.

```
## Ratio_Prot missing value overview:
```

```
## Ratio_Phos_MS missing value overview:
```

# Normalization

Normalization plots show how the data changes pre and post
normalization. If no normalization method was chosen, only one plot will
be generated. Additional normalization visualizations - MA plots,
Relative Log Expression plots, and QQ plots - can be found in the
associated image download folder.

# PCA Plot

Principal Component Analysis is a useful tool for assessing
similarity among factors of a high dimensional data set.

# UMAP

UMAP, or Uniform Manifold Approximation and Projection, is used for
dimensionality reduction and data visualization. It is commonly compared
to its predecessor, t-SNE or t-distributed Stochastic Neighbor
Embedding, but is generally much faster.

# MD Plots

Mean-Difference plots are used to visualize log-intensity ratios, or
‘differences’, versus log-intensity averages, or ‘means’.

# Correlation Plots

The correlation plots show correlation values among the groups of the
data set. The legend on the right shows the color scale over the range
of correlation values. Please note that the correlation plots are
assessing similarity over overall sample intensities.

# Heatmap

This heatmap shows an overview of the relative feature intensity
across the dataset(s) before linear modeling.

# Batch Correction PCA

These PCA plots are generated for each covariate included in the
model design. The batch corrected PCA is generated using an approximated
version of the dataset adjusted based on each covariate individually,
using the `removeBatchEffect()` function in the
`limma` package.

# Volcano Plots

The volcano plots are generated based on the options selected for the
linear model. Each contrast (or if contrasts are not selected each
group) has its own correlating volcano plot. The volcano plots show log
fold change values on the x-axis and log p-values or adjusted p-values
on the y-axis.

### Adjusted P-Value

### P-Value

# Differential Heatmaps

The differential heatmaps are based on the output of the limma linear
model. Each contrast or group has a corresponding heatmap, which is
subset by the top 50 most variable rows/genes.

# Enrichment

## Parameters

### Species

The selected species that correlated with the data input.

```
## mouse
```

### Enrichment Type

Either ranked or unranked enrichment analysis.

```
## Ranked Enrichment Analysis
```

```
## Gene Lists:
```

```
## List of 3
##  $ Ratio_Prot_GSEA    :List of 1
##   ..$ Fentanyl-Saline: Named num [1:9228] 3.26 3.15 3.13 2.81 2.7 ...
##   .. ..- attr(*, "names")= chr [1:9228] "Rbm48" "Ctdnep1" "Map3k20" "Dicer1" ...
##  $ Ratio_Phos_MS_GSEA :List of 1
##   ..$ Fentanyl-Saline: Named num [1:3432] 6.13 4.76 4.15 4.04 3.95 ...
##   .. ..- attr(*, "names")= chr [1:3432] "Setd1a" "Lmna" "Tanc2" "Ythdc1" ...
##  $ Ratio_Phos_MS_PKSEA:List of 1
##   ..$ Fentanyl-Saline: Named num [1:12333] 6.13 4.76 4.15 4.04 3.95 ...
##   .. ..- attr(*, "names")= chr [1:12333] "E9PYH6;S1402" "P48678;S414" "A2A690;S1476" "E9Q5K9;S146" ...
## NULL
```

```
## Enrichmed Pathways Present?
```

```
## Ratio_Prot_Fentanyl-Saline MOUSE_Reactome : TRUE 
## Ratio_Prot_Fentanyl-Saline MOUSE_GOBP : TRUE 
## Ratio_Prot_Fentanyl-Saline MOUSE_GOCC : TRUE 
## Ratio_Prot_Fentanyl-Saline MOUSE_GOMF : TRUE 
## Ratio_Prot_Fentanyl-Saline MOUSE_WikiPathways : TRUE 
## Ratio_Prot_Fentanyl-Saline MOUSE_MOMENTABiocycEXP : TRUE 
## Ratio_Phos_MS_Fentanyl-Saline MOUSE_Reactome : TRUE 
## Ratio_Phos_MS_Fentanyl-Saline MOUSE_GOBP : TRUE 
## Ratio_Phos_MS_Fentanyl-Saline MOUSE_GOCC : TRUE 
## Ratio_Phos_MS_Fentanyl-Saline MOUSE_GOMF : TRUE 
## Ratio_Phos_MS_Fentanyl-Saline MOUSE_WikiPathways : TRUE 
## Ratio_Phos_MS_Fentanyl-Saline MOUSE_MOMENTABiocycEXP : TRUE
```

## Dot Plot

The dot plot depicts the enrichment scores (p-values) via color-coded
points, and gene ratio as position relative to the x-axis. Gene count is
represented by the size of the point.

# S-Score Integration

### S-Score Integration: Ratio\_Prot\_Ratio\_Phos\_MS\_Fentanyl-Saline

## S-Score Enrichment

### Dot Plot

The dot plot depicts the enrichment scores (p-values) via color-coded
points, and gene ratio as position relative to the x-axis. Gene count is
represented by the size of the point.

# PCSF

```
## PCSF Datasets/Contrasts Generated: Ratio_Prot_Fentanyl-Saline; Ratio_Phos_MS_Fentanyl-Saline; Sscore_Ratio_Prot_Ratio_Phos_MS_Fentanyl-Saline
```

### Node Network: Sscore\_Ratio\_Prot\_Ratio\_Phos\_MS\_Fentanyl-Saline

visNetwork

### Influential Network: Sscore\_Ratio\_Prot\_Ratio\_Phos\_MS\_Fentanyl-Saline

visNetwork

## PCSF Enrichment

### PCSF Enriched: Sscore\_Ratio\_Prot\_Ratio\_Phos\_MS\_Fentanyl-Saline

### Enriched Network: Sscore\_Ratio\_Prot\_Ratio\_Phos\_MS\_Fentanyl-Saline

visNetwork

# Parameters

```
## File Name(s):  Ratio_Prot; Ratio_Phos_MS 
##  File Format(s):  ProteinGroups; PhosphoSites 
##  Species:  mouse 
##  Data Cleaning, Filtering, & Transformation: 
##       Normalization:  loess 
##       Log Transformation:  TRUE 
##       Percent Zeros Filter:  30 % 
##       Missing Value Imputation:  FALSE 
##       Uniprot Annotation:  TRUE 
##       Group By:  Drug 
##  Data Modeling: 
##       Contrasts:  Fentanyl-Saline 
##       Linear Model:  ~ 0 + Drug + Session 
##  Enrichment Performed on Linear Model:  TRUE 
##       Enrichment Algorithm:  ranked
```

# Session Info

See information about the system that generated this report
below.

```
## R version 4.4.1 (2024-06-14)
## Platform: x86_64-apple-darwin20
## Running under: macOS Sonoma 14.6.1
## 
## Matrix products: default
## BLAS:   /System/Library/Frameworks/Accelerate.framework/Versions/A/Frameworks/vecLib.framework/Versions/A/libBLAS.dylib 
## LAPACK: /Library/Frameworks/R.framework/Versions/4.4-x86_64/Resources/lib/libRlapack.dylib;  LAPACK version 3.12.0
## 
## locale:
## [1] en_US.UTF-8/en_US.UTF-8/en_US.UTF-8/C/en_US.UTF-8/en_US.UTF-8
## 
## time zone: America/Los_Angeles
## tzcode source: internal
## 
## attached base packages:
## [1] stats     graphics  grDevices utils     datasets  methods   base     
## 
## other attached packages:
##  [1] kableExtra_1.4.0    pathview_1.44.0     NormalyzerDE_1.22.0 Biobase_2.64.0      BiocGenerics_0.50.0 lubridate_1.9.3    
##  [7] forcats_1.0.0       stringr_1.5.1       dplyr_1.1.4         purrr_1.0.2         readr_2.1.5         tidyr_1.3.1        
## [13] tibble_3.2.1        ggplot2_3.5.1       tidyverse_2.0.0     shiny_1.9.1        
## 
## loaded via a namespace (and not attached):
##   [1] fs_1.6.4                    matrixStats_1.4.1           bitops_1.0-8                enrichplot_1.24.2          
##   [5] fontawesome_0.5.2           httr_1.4.7                  RColorBrewer_1.1-3          doParallel_1.0.17          
##   [9] Rgraphviz_2.48.0            dynamicTreeCut_1.63-1       tippy_0.1.0                 tools_4.4.1                
##  [13] backports_1.5.0             utf8_1.2.4                  R6_2.5.1                    DT_0.33                    
##  [17] uwot_0.2.2                  lazyeval_0.2.2              GetoptLong_1.0.5            sp_2.1-4                   
##  [21] withr_3.0.2                 gridExtra_2.3               preprocessCore_1.66.0       cli_3.6.3                  
##  [25] textshaping_0.4.0           pacman_0.5.1                Cairo_1.6-2                 scatterpie_0.2.4           
##  [29] shinyjs_2.1.0               topGO_2.56.0                labeling_0.4.3              sass_0.4.9                 
##  [33] KEGGgraph_1.64.0            bs4Dash_2.3.4               robustbase_0.99-4           proxy_0.4-27               
##  [37] ggridges_0.5.6              systemfonts_1.1.0           commonmark_1.9.1            yulab.utils_0.1.7          
##  [41] gson_0.1.0                  svglite_2.1.3               DOSE_3.30.4                 R.utils_2.12.3             
##  [45] limma_3.60.4                rstudioapi_0.16.0           RSQLite_2.3.7               FNN_1.1.4                  
##  [49] gridGraphics_0.5-1          visNetwork_2.1.2            generics_0.1.3              shape_1.4.6.1              
##  [53] crosstalk_1.2.1             vroom_1.6.5                 car_3.1-2                   zip_2.3.1                  
##  [57] dendextend_1.17.1           GO.db_3.19.1                Matrix_1.7-0                fansi_1.0.6                
##  [61] S4Vectors_0.42.1            abind_1.4-5                 bsicons_0.1.2               R.methodsS3_1.8.2          
##  [65] lifecycle_1.0.4             yaml_2.3.10                 carData_3.0-5               SummarizedExperiment_1.34.0
##  [69] qvalue_2.36.0               SparseArray_1.4.8           grid_4.4.1                  blob_1.2.4                 
##  [73] promises_1.3.0              crayon_1.5.3                miniUI_0.1.1.1              lattice_0.22-6             
##  [77] ComplexUpset_1.3.3          cowplot_1.1.3               KEGGREST_1.44.1             magick_2.8.4               
##  [81] pillar_1.9.0                knitr_1.48                  ComplexHeatmap_2.20.0       fgsea_1.30.0               
##  [85] GenomicRanges_1.56.1        boot_1.3-30                 rjson_0.2.23                codetools_0.2-20           
##  [89] fastmatch_1.1-4             glue_1.8.0                  ggvenn_0.1.10               ggfun_0.1.6                
##  [93] data.table_1.16.0           vcd_1.4-12                  treeio_1.28.0               vctrs_0.6.5                
##  [97] png_0.1-8                   org.Mm.eg.db_3.19.1         colourvalues_0.3.9          gtable_0.3.6               
## [101] cachem_1.1.0                xfun_0.48                   openxlsx_4.2.7              S4Arrays_1.4.1             
## [105] mime_0.12                   tidygraph_1.3.1             pheatmap_1.0.12             iterators_1.0.14           
## [109] statmod_1.5.0               nlme_3.1-164                ggtree_3.12.0               bit64_4.0.5                
## [113] GenomeInfoDb_1.40.1         influential_2.2.9           bslib_0.8.0                 irlba_2.3.5.1              
## [117] colorspace_2.1-1            DBI_1.2.3                   nnet_7.3-19                 mnormt_2.1.1               
## [121] DESeq2_1.44.0               tidyselect_1.2.1            bit_4.0.5                   compiler_4.4.1             
## [125] curl_6.0.0                  httr2_1.0.5                 graph_1.82.0                SparseM_1.84-2             
## [129] expm_1.0-0                  xml2_1.3.6                  GeneTonic_2.8.0             DelayedArray_0.30.1        
## [133] plotly_4.10.4               shadowtext_0.1.4            colourpicker_1.3.0          scales_1.3.0               
## [137] lmtest_0.9-40               DEoptimR_1.1-3              psych_2.4.6.26              rappdirs_0.3.3             
## [141] digest_0.6.37               rmarkdown_2.28              XVector_0.44.0              htmltools_0.5.8.1          
## [145] pkgconfig_2.0.3             MatrixGenerics_1.16.0       highr_0.11                  PCSF_0.99.1                
## [149] fastmap_1.2.0               rlang_1.1.4                 GlobalOptions_0.1.2         htmlwidgets_1.6.4          
## [153] UCSC.utils_1.0.0            farver_2.1.2                jquerylib_0.1.4             zoo_1.8-12                 
## [157] jsonlite_1.8.9              BiocParallel_1.38.0         GOSemSim_2.30.2             R.oo_1.26.0                
## [161] RCurl_1.98-1.16             magrittr_2.0.3              ggplotify_0.1.2             GenomeInfoDbData_1.2.12    
## [165] patchwork_1.2.0             munsell_0.5.1               Rcpp_1.0.13                 ape_5.8                    
## [169] shinycssloaders_1.1.0       viridis_0.6.5               stringi_1.8.4               rintrojs_0.3.4             
## [173] ggraph_2.2.1                zlibbioc_1.50.0             MASS_7.3-60.2               plyr_1.8.9                 
## [177] org.Hs.eg.db_3.19.1         parallel_4.4.1              ggrepel_0.9.6               Biostrings_2.72.1          
## [181] graphlayouts_1.1.1          splines_4.4.1               BH_1.84.0-0                 hms_1.1.3                  
## [185] circlize_0.4.16             locfit_1.5-9.10             ranger_0.16.0               igraph_2.0.3               
## [189] ggpubr_0.6.0                ggsignif_0.6.4              markdown_1.13               reshape2_1.4.4             
## [193] stats4_4.4.1                XML_3.99-0.17               evaluate_0.24.0             laeken_0.5.3               
## [197] tzdb_0.4.0                  foreach_1.5.2               tweenr_2.0.3                httpuv_1.6.15              
## [201] VIM_6.2.2                   backbone_2.1.4              polyclip_1.10-7             clue_0.3-65                
## [205] ggforce_0.4.2               broom_1.0.6                 xtable_1.8-4                e1071_1.7-14               
## [209] tidytree_0.4.6              rstatix_0.7.2               later_1.3.2                 class_7.3-22               
## [213] viridisLite_0.4.2           ragg_1.3.2                  clusterProfiler_4.12.6      aplot_0.2.3                
## [217] memoise_2.0.1               AnnotationDbi_1.66.0        IRanges_2.38.1              cluster_2.1.6              
## [221] timechange_0.3.0            shinyWidgets_0.8.6          shinyAce_0.4.2
```
